# Synthesis of 5-Fluorouracil Polymer Conjugate and ^19^F NMR Analysis of Drug Release for MRI Monitoring

**DOI:** 10.3390/polym15071778

**Published:** 2023-04-03

**Authors:** Laila M. Alhaidari, Sebastian G. Spain

**Affiliations:** 1 Department of Chemistry, Faculty of Science, University of Majmaah, Majmaah 11952, Saudi Arabia; 2 Department of Chemistry, Dainton Building, University of Sheffield, Sheffield S3 7HF, UK

**Keywords:** ^19^F MRI, 5-fluorouracil, self-condensing vinyl polymerization, reversible addition-fragmentation chain transfer

## Abstract

To monitor the release of fluorinated drugs from polymeric carriers, a novel ^19^F MRI enzyme-responsive contrast agent was developed and tested. This contrast agent was prepared by conjugation of 5-fluorouracil (5-FU) to hyperbranched poly(*N*,*N*-dimethylacrylamide) (HB-PDMA) via an enzyme-degradable peptide linker. Due to the different molecular sizes, the release of 5-FU from the 5-FU polymer conjugate resulted in a sufficiently substantial difference in spin-spin *T*_2_ ^19^F NMR/MRI relaxation time that enabled differentiating between attached and released drug states. The 5-FU polymer conjugate exhibited a broad signal and short *T*_2_ relaxation time under ^19^F NMR analysis. Incubation with the enzyme induced the release of 5-FU, accompanied by an extension of *T*_2_ relaxation times and an enhancement in the ^19^F MRI signal. This approach is promising for application in the convenient monitoring of 5-FU drug release and can be used to monitor the release of other fluorinated drugs.

## 1. Introduction

Real-time monitoring of drug release is of vital importance, not only to ensure site-specific drug delivery but also to improve therapeutic efficiency and avoid inappropriate drug dosage. Magnetic resonance imaging (MRI) [1], based on ^1^H is perhaps one of the most used imaging modalities for noninvasive diagnostic applications. MRI can visualize deep regions of the body without the use of harmful ionizing radiation or radioactive tracers [2,3]. Nevertheless, MRI is a relatively insensitive imaging technique, and the use of contrast agents such as gadolinium chelates [4,5] and iron oxide nanoparticles [6,7,8] is often required for sufficient visualization. These contrast agents work by altering the relaxation time of nearby water protons, thereby providing contrast in the image. The contrast generated, however, is often limited as a result of the large background signal arising from intrinsic ^1^H nuclei found in mammalian tissues. Furthermore, it is extremely challenging to obtain quantitative information from images employing ^1^H MRI contrast agents.

To overcome the aforementioned challenges, there has been intense interest in the development of contrast agents that can complement ^1^H and are based on biologically rare, magnetically active nuclei such as ^19^F. In the human body, ^19^F is found only in the teeth and bones, where it is found throughout in the form of immobilized salts in trace amounts, resulting in a fast spin–spin relaxation time (*T*_2_) and a background signal that is much lower than conventional NMR/MRI detection limits [9]. ^19^F exhibits a number of properties that make it an excellent candidate for analysis by NMR, including 100% natural isotopic abundance, high gyromagnetic ratio of 40.08 MHz T^−1^, high sensitivity (83% of ^1^H), and a large chemical shift range (~300 ppm) [10]. Moreover, the NMR signal intensity is directly proportional to the fluorine content, which makes it a good candidate for quantitative applications such as the real-time monitoring of drug delivery [11], tumor oxygenation studies [12], and cell tracking [13,14].

Numerous fluorinated compounds ranging from small molecules [15] to polymers [16,17] have been proposed as ^19^F MRI contrast agents. A series of recent studies have indicated that polymeric contrast agents are particularly attractive for this purpose and, accordingly, several classes of polymeric agents including linear polymers [18,19], star polymers [20,21], hyperbranched polymers (HBPs) [22,23], and polymer nanogels [24,25] have been developed. Among this wide range of polymeric structures, HBPs are potentially promising candidates for use as ^19^F MRI contrast agents [26,27,28,29]. Due to their constrained 3D shape, a high concentration of highly separated ^19^F nuclei can be achieved, thus maintaining high segmental mobility. This in turn leads to efficient averaging of the dipole–dipole coupling and satisfactory ^19^F MRI image quality. Furthermore, there are a large number of end groups available for conjugating drug molecules or complementary imaging moieties.

More recently, significant efforts have focused on the development of stimuli-responsive ^19^F MRI contrast agents. These contrast agents remain invisible until encountering a certain stimulus such as pH [20,30,31], redox [31,32], or enzyme [33,34,35,36,37]. In particular, a number of interesting strategies have been proposed to obtain an enzyme-responsive ^19^F MRI switch. The mechanism of activation is mainly based on either a chemical shift change [38,39] or a signal intensity switch induced by paramagnetic relaxation enhancement (PRE) upon enzymatic cleavage [33,34,35,36,37]. Despite the success in the use of the chemical shift approach to detect enzyme activity [38,39,40], the approach is dependent on the magnitude of the chemical shift change being sufficiently large, which is often a limiting factor. In contrast, a later technique based on the PRE effect seems very promising. For example, Mizukami et al. [36] reported a switchable contrast agent, where a Gd-based chelate was linked to a trifluoromethoxy benzyl group via a degradable peptide. Initially, the Gd(III) ion conveys an intramolecular PRE effect upon the fluorine nuclei, which leads to the *T*_2_ relaxation time shortening to the point of being undetectable. Upon cleavage of the peptide, the intramolecular PRE on the ^19^F nuclei is effectively negated, resulting in *T*_2_ lengthening and, therefore, significant enhancement of the signal.

**Scheme 1:**
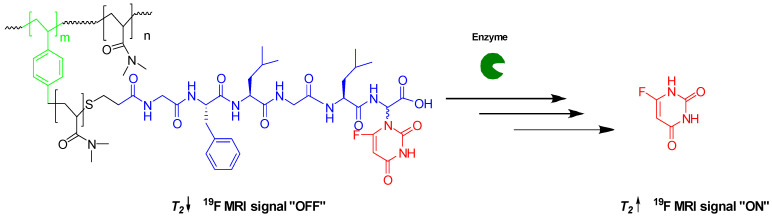
OFF/ON ^19^F MRI switch induced by the enzymatic cleavage of 5-FU.

To the best of our knowledge, no enzyme-responsive polymeric-based contrast agents have been developed. Here, we report the synthesis of a new ^19^F contrast agent based on a HBP–peptide–fluorinated drug conjugate for monitoring the release of the fluorinated drug. 5-Fluorouracil (5-FU) was specifically selected as the model fluorinated drug for this study because of its ^19^F NMR detectable signal. The hypothesis is that the 5-FU release will induce a change in *T*_2_, detectable by ^19^F NMR, that is sufficiently substantial to allow differentiation between attached and released drug states (Scheme 1). Molecular size and, hence, ^19^F nuclei motion are keys to obtaining optimal ^19^F MRI contrast. ^19^F nuclei within the 5-FU polymer conjugate experience slow molecular motion that leads to a significantly shortened *T*_2_ relaxation time. Since the signal line width is inversely proportional to the *T*_2_ relaxation time, a short *T*_2_ results in a broad ^19^F NMR signal. Incubation of the 5-FU polymer conjugate with the enzyme induces the release of 5-FU, accompanied by an increase in the *T*_2_ relaxation times and, hence, a sharp ^19^F NMR signal.

To achieve this, a HBP that is covalently conjugated to a biodegradable oligopeptide with 5-FU in its α C-terminal glycine residue was synthesized. The selection of this oligopeptide was based upon previously reported work by Putnam and Kopecek [41], based on its known degradation ability, while the selection of the HBP was based on its shape persistence for averaging ^19^F dipole–dipole coupling along with its high density of thiocarbonylthio end groups for 5-FU prodrug conjugation. The HBP was synthesized by reversible addition–fragmentation chain transfer mediated self-condensing vinyl polymerization (RAFT-SCVP). After polymerization, the thiocarbonylthio groups residing at the chain ends were reduced to the corresponding thiols, which were trapped in situ by the vinyl oligopeptide followed by the attachment of the 5-FU prodrug. The ability to monitor the release of 5-FU from the polymer conjugate using ^19^F NMR was then examined in the presence of the S9 fraction.

## 2. Materials and Methods

### 2.1. Material

*N*,*N*-Dimethylacrylamide (DMA, 99%), 4,4′-azobis(4-cyanovaleric acid) (ACVA, ≥98%), benzyl 1H-pyrrole-1-carbodithioate (BPC, 97%), Fmoc-Gly-Wang resin (100–200 mesh, capacity: 0.56 mmol g^−1^), Fmoc amino acids, triisopropylsilane (98%), propylamine (≥99%), phenyldimethylphosphine (99%), DMF (peptide synthesis grade quality), magnesium chloride hexahydrate (99%), and pentafluorophenol (≥99%) were purchased from Sigma-Aldrich. 1-[bis(Dimethylamino)methylene]-1H-benzotriazolium hexafluorophosphate 3-oxide (HBTU, 98%), *N*,*N*-diisopropylethylamine (DIPEA, 99%), and *N*,*N*-dicyclohexylcarbodiimide (DCC, 99%) were purchased from Alfa Aesar. 4-Dimethylaminopyridine (99%), 1,4-dioxane (extra dry, 99.8%), and trifluoroacetic acid (TFA, 99.5%) were purchased from Acros Organics. All other solvents were analytical grade and purchased from Fisher Scientific. DMA was vacuum distilled, and ACVA was recrystallized twice from methanol prior to use. All other chemicals were used as received, without any purification. 4-Vinylbenzyl *N*-pyrrole carbodithioate (VBPC) was synthesized and purified in-house as previously reported by Rimmer et al. [42]. 

### 2.2. Nuclear Magnetic Resonance (NMR)

^1^H and ^13^C NMR analysis were performed on either a Bruker AV400 or Bruker AVIII HD 400 spectrometer at room temperature. Chemical shifts of spectrums were estimated in ppm relative to the residual solvent peak, and the NMR spectra were examined using Topspin 3.0 NMR software. Multiplicities are described using the following abbreviations: s = singlet, br = broad, d = doublet, t = triplet, and m = multiplet.

^19^F NMR spectra were acquired at 376.5 MHz without ^1^H decoupling on a Bruker AVIII400 spectrometer fitted with a 5 mm auto-tunable broadband (BBFO) probe. The relaxation delay was 25 s, and the acquisition time was 1.1 s. Data were collected using a spectral width of 59 kHz and 16–46 scans. TFA was used as an internal standard reference (−75.43 ppm).

^19^F spin–lattice relaxation times (*T*_1_) were measured using the standard inversion-recovery (IR) pulse sequence. The relaxation delay was 30 s, and the acquisition time was 1.1 s. Data were collected using a spectral width of 59 kHz and 32–128 scans. For each measurement, the recovery times were from 1 ms to 60 s, and 12 points were collected. *T*_1_ was then calculated by TopSpin 3.0 using area type fitting. A single-component exponential recovery fit was used (Equation (1)).

(1) $$I\left(\tau \right)=I\left(0\right)+P\;\exp\left(-\frac{\tau }{T_{1}}\right)$$

^19^F spin–spin relaxation times (*T*_2_) were measured using the Carr–Purcell–Meiboom–Gill (CPMG). The relaxation delay was 20 s, and the acquisition time was 1.1 s. Data were collected using a spectral width of 9.4 kHz and 128–512 scans. For each measurement, the delay times ranged from 8 ms to 1.2 s, and 12 points were collected. *T*_2_ was then calculated by TopSpin 3.0 using area type fitting. A single-component exponential decay fit was used (Equation (2)).

(2) $$I\left(\tau \right)=P\exp\left(-\frac{\tau }{T_{2}}\right)$$

### 2.3. Gel Permeation Chromatography (GPC)

Molecular weights and molecular weight distributions were determined using an Agilent 1260 Infinity GPC/SEC system. The GPC system was equipped with a refractive index (RI) detector. The molecular weights were estimated relative to near-monodisperse poly(methyl methacrylate) standards (2.14 × 10^6^–1.95 × 10^3^ g mol^−1^ range). DMF with 0.1% LiBr was used as the mobile phase at a flow rate of 1.0 mL min^−1^. Samples were prepared at a 1 mg mL^−1^ concentration using a solution of DMF, 0.1% LiBr, and 0.1% toluene as a marker reference. Samples were filtered through a 0.45 mm PTFE syringe before injection.

### 2.4. Dynamic Light Scatting (DLS)

Intensity-average size distributions were determined by Malvern Zetasizer software using a Malvern Zetasizer NanoZS Model ZEN 3600 instrument at a fixed angle of 173°. Measurements were performed at 25 °C on the polymer solutions in water with a concentration of 2 mg mL^−1^. Three measurements of approximately ten runs of ten seconds duration were made and averaged.

### 2.5. Transmission Electron Microscopy (TEM)

TEM studies were accomplished using a FEI Tecnai Spirit Microscope operating at an accelerating voltage of 100 kV. Samples were prepared by placing a droplet (5 μL) of the polymer solution (10 mg/ mL) on a glow-discharged, carbon-coated grid for approximately one minute. Samples were stained with a uranyl formate solution (5 μL of a 0.75% *w/w*).

### 2.6. Synthesis of Hyperbranched Poly(N,N-Dimethylacrylamide) (HB-PDMA)

For the synthesis of P3, DMA (30 mmol, 3 g), ACVA (0.12 mmol, 0.03 g), and VBPC (0.60 mmol, 0.16 g) were dissolved in anhydrous dioxane (6.79 mL). The yellow solution was then transferred into a Schlenk flask and degassed using three freeze–pump–thaw cycles before being backfilled with nitrogen. The flask was immersed in a water bath at 60 °C. After completion of polymerization, the crude polymer solution was precipitated in a 9:1 ratio of diethyl ether/acetone mixture. The precipitate was isolated by centrifugation (4500 rpm for 5 min). The polymer was then dialyzed against deionized water (membrane MWCO 3.5 kDa) for 48 h. A yellow solid was obtained after lyophilization. The characteristics of the polymers are listed in Table 1.

^1^H NMR (400 MHz, CDCl_3_) δ 7.75–7.65 (br, 2H), 7.17–6.83 (br, 4H), 6.36–6.31 (br, 2H), 5.19–5.01 (br, 1H), 3.28–2.75 (br. m., 6H), 2.77–2.03 (br. m., 1H), 2.03–1.07 (br. m., 2H). 

### 2.7. Synthesis of Vinyl-Modified Tetrapeptide (Gly-Leu-Phe-Gly)

The peptide was synthesized via the Fmoc solid phase procedure [43], using Fmoc-Gly-Wang resin (100–200 mesh, substitution = 0.56 mmol g^−1^). *N*-Fmoc amino acids: Fmoc-Leu-OH, Fmoc-Phe-OH, and Fmoc-Gly-OH were coupled in sequence to Fmoc-Gly-Wang resin (1 g) using HBTU as the coupling agent. Once Gly-Phe-Leu-Gly was synthesized and Fmoc on the *N*-terminus was deprotected, an excess amount of the acrylic acid (201.76 mg, 2.80 mmol) activated with HBTU (1040.63 mg, 2.70 mmol) in the presence of DIPEA (723.74 mg, 5.6 mmol) was added, followed by washing with DMF and then DCM. After cleavage using [TFA/H_2_O/TIPS 9.0/0.5/0.5], the crude peptide was precipitated into cold ether. The precipitate was isolated by centrifugation (4500 rpm for 5 min) and dried under vacuum at room temperature to a white powder material with a yield of 87%. The purity was found to be 81%, as determined by reverse phase HPLC (column: Waters XBridge C18 250 × 4.6 mm) with a linear gradient of 5 to 95% acetonitrile (with 0.1% formic acid) over 20 min at a flow rate of 1 mL min^−1^ (Figure S6).

^1^H NMR (400 MHz, DMSO) δ 12.54 (s, 1H), 8.33 (t, *J* = 5.7 Hz, 1H), 8.23–7.94 (overlapped, 3H), 7.27–7.21 (m, 4H), 7.21–7.15 (m, 1H), 6.28 (dd, *J* = 16.9, 10.2 Hz, 1H), 6.09 (dd, *J* = 17.1, 2.1 Hz, 1H), 5.60 (dd, *J* = 10.2, 2.1 Hz, 1H), 4.58–4.52 (m, 1H), 4.34 (q, *J* = 7.7 Hz, 1H), 3.87–3.61 (m, 4H), 3.04 (dd, *J* = 13.8, 4.2 Hz, 1H), 2.78 (dd, *J* = 13.8, 9.5 Hz, 1H), 1.66–1.56 (m, 1H), 1.49 (t, *J* = 7.2 Hz, 2H), 0.87 (dd, *J* = 18.4, 6.5 Hz, 6H).

ESI–MS: expected m/z: 446.2, experimental m/z: 447.2 (M^+^ + H)

### 2.8. Synthesis of Leu-Gly(5-FU)

*N*-(Carbobenzyloxy)-L-leucyl-2-(5-fluorouracil-1-y1)-L,D-glycine (Cbz-Leu-Gly(5-FU)) was synthesized and purified in-house, as previously reported by Putnam and Kopecek [41]. The deprotection of Cbz was accomplished using 1,4-cyclohexadiene following the procedure described by Schacht [44]. A solution of Cbz-Leu-Gly(5-FU) (420 mg, 933 mmol) in dry ethanol (40 mL) was added to a round-bottomed flask equipped with a magnetic stirring bar. After purging the mixture with N_2_ for 30 min, 10% Pd/C (420 mg), freshly distilled 1,4-cyclohexadiene (806 mg, 6.05 mmol), and acetic acid (0.11 mL) were added. The reaction progress was monitored by TLC using DCM/MeOH 95:5 as an eluent. The crude reaction mixture was filtered through Celite and the residue in the filter was rinsed with methanol (2 × 50 mL) containing 0.01% acetic acid. The filtrate solvent was removed by rotary evaporator under vacuum. The conversion was about 79% as calculated by ^1^H NMR. The diastereomers were purified directly by preparative reverse phase HPLC (Column: C18 Waters XBridge 2 µm OBD 19 × 250 mm) using a gradient of 5 to 15% acetonitrile (with 0.1% TFA) over 20 min. Both fractions eluting from 6.104 to 7.011 min and from 8.147 to 9.210 min were collected. The first fraction was assigned as **9a** (L,L), while the second fraction was assigned as **9a** (L,D). To remove TFA counterions from the isolated dipeptide, 50 mg of either **9a** or **9b** was dissolved in 50 mL of 5 mM HCl and subsequently lyophilized. The process was repeated twice to ensure the complete elimination of TFA.

9a (L,L):

[α]^25^_D_ = +81.0° (c = 1, H_2_O) (Lit [α]^25^_D_ = +94.0° (c = 1, H_2_O)) [41]

^1^H NMR (400 MHz, DMSO) δ 12.07 (d, *J* = 3.2 Hz, 1H), 9.82 (d, *J* = 7.9 Hz, 1H), 8.19 (s, 3H), 8.15 (d, *J* = 6.6 Hz, 1H), 6.22 (d, *J* = 7.8 Hz, 1H), 3.95 (br, 1H), 1.73–1.62 (m, 1H), 1.59 (t, *J* = 7.0 Hz, 2H), 0.92 (dd, *J* = 10.7, 6.4 Hz, 6H).

^19^F NMR (377 MHz, DMSO) δ −169.81.

^13^C NMR (101 MHz, DMSO) δ 170.94, 167.21, 157.66, 149.25, 138.20, 140.49, 130.01, 64.21, 51.06, 24.04, 23.14, 22.27.

ESI–MS: expected m/z: 316.12, experimental m/z: 317.1 (M^+^ + H)

Elemental analysis calc.: C = 39.06%, H = 4.22%, N = 13.02%. Found: C = 37.11%, H = 5.88%, N = 13.78%.

9b (L,D):

[α]^25^_D_ = −74.0° (c = 1, H_2_O) (Lit [α]^25^_D_ = −70.6° (c = 1, H_2_O)) [41]

^1^H NMR (400 MHz, DMSO) δ 12.08 (d, *J* = 4.7 Hz, 1H), 9.88 (d, *J* = 7.3 Hz, 1H), 8.23 (s, 3H), 8.16 (d, *J* = 6.7 Hz, 1H), 6.26 (d, *J* = 7.3 Hz, 1H), 4.02 (br, 1H), 1.62–1.54 (m, 1H), 1.55–1.48 (m, 2H), 0.83 (d, *J* = 5.6 Hz, 6H).

^19^F NMR (377 MHz, DMSO) δ −169.62 (s).

^13^C NMR (101 MHz, DMSO) δ 170.88, 167.18, 157.56, 149.26, 139.35, 140.48, 129.92, 63.99, 50.91, 23.87, 23.00, 22.37.

ESI–MS: expected m/z: 316.12, experimental m/z: 317.1 (M^+^ + H)

Elemental analysis calc.: C = 39.06%, H = 4.22%, N = 13.02%. Found: C = 38.58%, H = 5.73%, N = 14.94%.

### 2.9. Synthesis of HB-PDMA-Gly-Leu-Phe-Gly 

A solution of P3 (0.019 mmol, 500 mg, 25.5 kDa, *Ð* = 3.77) in DMF (3 mL) was added to a round-bottomed flask equipped with a magnetic stirring bar. After purging the mixture with N_2_ for 15 min, propylamine (0.537 mmol, 31.7 mg) and Gly-Phe-Leu-Gly (0.537 mmol, 239.5 mg) were added. The reaction mixture was purged for another 15 min, and phenyldimethylphosphine (0.644 mmol, 88.9 mg, 91 µL) was then added. The reaction mixture was stirred at room temperature overnight. The conjugate was isolated by precipitation in diethyl ether/acetone 9:1 followed by dialysis against deionized water for 48 h. A white solid was obtained after lyophilization, with a yield of 412 mg (85%).

^1^H NMR (400 MHz, D_2_O) δ 7.36–7.14 (br. m, 5H), 7.28–6.77 (br, 4H), 4.59–4.47 (br. m, 1H), 4.27 (s, 1H), 3.79 (s, 4H), 3.27–2.71 (m, 6H), 2.77–2.03 (br. m, 1H), 2.03–1.07 (br. m, 1H), 0.80 (dd, *J* = 22.2, 5.6 Hz, 6H).

### 2.10. Activation of HB-PDMA-Gly-Leu-Phe-Gly Carboxylic Group

A mixture of HB-PDMA-Gly-Phe-Leu-Gly (0.016 mmol, 483 mg), 4-(dimethylamino)pyridine (0.021 mmol, 2.5 mg), pentafluorophenol (0.207 mmol, 38 mg), and DMF (2 mL) were added to a round-bottomed flask equipped with a magnetic stirring bar. After purging the mixture with N_2_ for 20 min, a solution of DCC (0.207 mmol, 42.8 mg) in DMF (1 mL) was added. The reaction mixture was stirred for 1 h at 0 °C and for another 20 h at room temperature. The crude reaction mixture was filtered to remove the insoluble dicyclohexylurea precipitates. The filtrate was concentrated using a rotary evaporator and purified by precipitation in diethyl ether/acetone (9:1), three times. A white solid material was obtained after drying under vacuum at room temperature, with a yield of 355 mg (87%). 

^1^H NMR (400 MHz, DMSO) δ 7.28–7.09 (br. m, 5H), 7.14–6.73 (br. m, 4H), 5.57 (d, *J* = 8.0 Hz, 2H), 4.60–4.47 (br. m., 1H), 4.45–4.26 (br. m., 2H), 4.23–4.13 (br. m., 1H), 3.78–3.64 (br. m., 1H), 3.65–3.49 (br. m., 1H), 3.21–2.61 (br. m., 6H), 2.61–1.90 (br. m., 1H), 1.89–0.93 (br. m., 2H), 0.87 (dd, *J* = 19.4, 6.5 Hz, 6H).

^19^F NMR (377 MHz, DMSO) δ −153.11, −157.77, −162.49.

### 2.11. Synthesis of HB-PDMA-Gly-Leu-Phe-Gly-Leu-Gly(5-FU) 

A solution of HB-PDMA-Gly-Phe-Leu-Gly (0.015 mmol, 355 mg) in dry DMF was added to a round bottomed flask equipped with a magnetic stirring bar. After purging the mixture with N_2_ for 30 min, Leu-Gly(5-FU) (0.207 mmol, 89 mg) and *N*,*N*-diisopropylethylamine (0.414 mmol, 53 mg, 72 µL) were added. The reaction mixture was stirred at room temperature overnight. The reaction mixture was concentrated by rotary evaporator under vacuum and precipitated in diethyl ether. The 5-FU polymer conjugate was purified by dialysis against deionized water followed by lyophilization to give a white solid material, with a yield of 309 mg (81%).

^1^H NMR (400 MHz, D_2_O) δ 7.79 (d, J = 5.7 Hz, 1H), 7.36–7.07 (br. m., 1H), 7.32–6.73 (br. m., 4H), 5.89 (s, 1H), 4.60–4.46 (br. m., 1H), 4.41–4.33 (br. m., 1H), 4.30–4.23 (br. m., 1H), 4.23–4.16 (br. m., 1H), 3.94–3.69 (br. m., 4H), 3.71–3.51 (br. m., 2H), 3.32–2.71 (br. m., 6H), 2.71–2.26 (br. m., 1H), 1.18–1.77 (br. m., 2H), 0.91–0.67 (br. m., 12H).

*M_n_* = 22,000 Da, *Ð* = 2.59

### 2.12. Release of 5-FU from the Polymer Conjugate and Dipeptide Derivatives of 5-FU 

The release of 5-FU from the polymer conjugate, L,L, and L,D dipeptide derivatives of 5-FU, was investigated in a 5 mm NMR tube using TFA as an internal standard reference (−75.43 ppm). The appropriate amount of 5-FU was added to each sample (i.e., the polymer conjugate or the dipeptides) in a 1 mL volume of PBS/D_2_O (9:1) containing 3 mM MgCl_2_ at pH 7.4 to give a final concentration of 15 µM 5-FU. The S9 fraction from rat liver, corresponding to 1 µg, was then added, and the samples were immersed in a water bath at 37 °C for 24 h. 5-FU release was monitored using 1D ^19^F NMR along with ^19^F *T*_1_/*T*_2_. The assignment of the resonance signals for dipeptides and 5-FU was accomplished by comparing the chemical shifts with those of known compounds analyzed under the same conditions using TFA as an internal standard reference (75.43 ppm).

## 3. Results and Discussion

### 3.1. Synthesis of Hyperbranched Poly(N,N-dimethylacrylamide) (HB-PDMA)

**Scheme 2:**
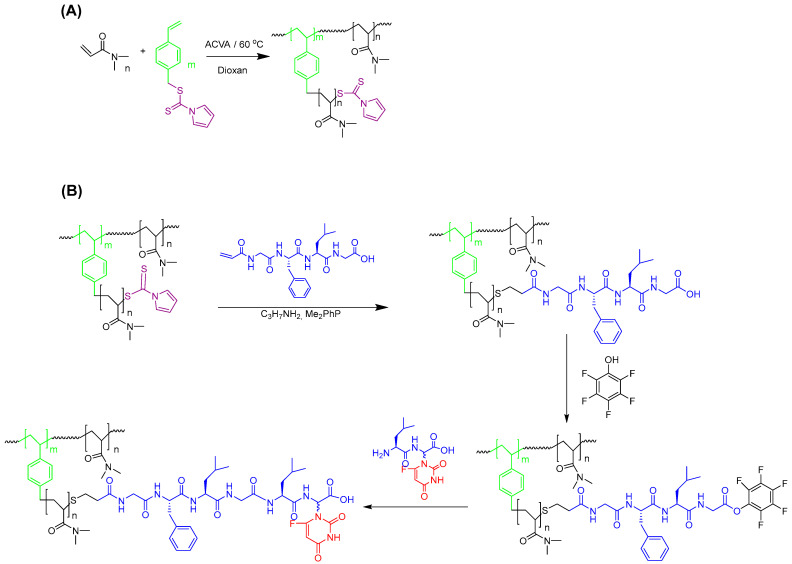
(**A**) The synthesis of hyperbranched poly(*N*,*N*-dimethylacrylamide) (HB-PDMA) via RAFT-SCVP. (**B**) Synthetic route to 5-FU polymer conjugate.

Hyperbranched poly(*N*,*N*-dimethylacrylamide) (HB-PDMA) was synthesized by RAFT-SCVP. RAFT-SCVP was particularly selected, as this elegant technique allows for the large-scale synthesis of HBPs from commercially available vinyl monomers in one pot with the aid of a chain transfer monomer (CTM) [45]. As illustrated in Scheme 2A, *N*,*N*-dimethylacrylamide (DMA) was copolymerized with 4-vinylbenzyl *N*-pyrrole carbodithioate (VBPC) in 1,4-dioxane as the solvent in the presence of 4,4′-azobis(4-cyanovaleric acid) (ACVA) as a radical initiator. VBPC is a typical CTM with a thiocarbonylthio group for the polymerization of both the styryl vinyl group and DMA. The selection of VBPC was based on the successful synthesis of soluble hyperbranched polyacrylamides with a high monomer conversion [42,46,47,48]. The feed ratio of [DMA]:[VBPC] (γ) was kept constant at 50:1, while the monomer concentration in the solvent varied from 10 to 50 wt% for polymers labeled P1 to P5. The ratio of ACVA to VBPC was kept low (1:0.2) to ensure high chain-end fidelity for the conjugation of the 5-FU prodrug. The results of copolymerization are summarized in Table 1.

**Figure 1:**
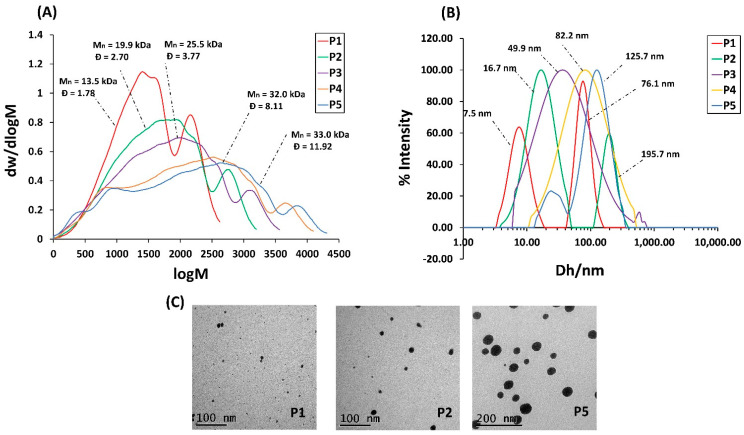
(**A**) Molecular weight distribution of HBPs derived from DMF GPC. (**B**) Particle size distribution of HBPs derived from DLS in water operated at 25 °C. (**C**) TEM images of HBPs stained by uranyl formate.

Despite the high DMA conversion, no gel was observed, even for high concentration systems. The risk of gelation, however, increases with concentration as the reaction progresses. For example, P5 turned completely to gel after 4 h (more than 92% conversion), possibly due to cross-linking via the bimolecular termination of polymeric radicals. Shorter reaction times were requested for high concentration systems to form soluble HBPs. The number average molecular weight *M_n_* and dispersity *Ð* were determined using gel permeation chromatography (GPC) relative to linear polymethyl methacrylate (PMMA) standards. With the increase in the reaction concentrations from 10 to 50 wt%, the *M_n_* of HBPs varied from 13.5 to 33.0 kDa with high *Ð* in the range of 1.8–11.9. It should be emphasized that the *M_n_* values obtained from GPC are underestimated, and the actual *M_n_* values are expected to be much higher as HBPs exhibit a smaller hydrodynamic volume than their linear analogues. The molecular weight distributions shown in Figure 1A are broad with multiple components, as expected for HBPs synthesized by RAFT-SCVP. These distributions have slight shoulders, suggesting the presence of lower molecular weight fractions. These low molecular weight fractions appear to be less prominent in the higher concentration samples, where linking reactions are more likely compared to lower concentration systems.

The hydrodynamic size of HBPs in water was measured using dynamic light scattering (DLS) operated at 25 °C (Figure 1B). The hydrodynamic size varied from 11 nm to 122 nm with increasing concentration. P1 and P2 show bimodal distribution. The main distribution may represent individual particles, while the minor one represents the aggregation. The particle size was also measured by transmission electron microscopy (TEM), and the results were compared with DLS results (Table S2). Figure 1C shows TEM images of P1, P2, and P5 stained with uranyl formate. The HBPs diameters assessed from TEM confirmed the trend obtained from DLS; however, they are generally much smaller. This is somewhat expected since DLS is an intensity-based technique and shows more emphasis on the larger objects of the distribution, while TEM is a number-based technique and shows stronger emphasis on the smallest objects of the distribution.

The *DB* was calculated using Equation (3), where *D* and *L* represent the mole fraction of the dendritic and linear units, respectively. The *D* value was determined by integrating one proton at 6.83–7.17 ppm, corresponding to the styryl proton, while *L* was calculated by integrating the DMA methyl proton at 2.75–3.28 ppm.

(3) $$DB=\frac{2D}{2D+L}$$

Despite the variation of the concentration, the same final *DB* of 0.038 was obtained. This is not surprising since *DB* is mainly dependent on γ, which was kept constant in this report. The calculated *DB* is in a good agreement with the theoretical value of 0.039 (Table 1).

The retention of the thiocarbonylthio group, required for 5-FU prodrug conjugation, was calculated using ^1^H NMR spectroscopy by comparing the ratio of the integrals for the pyrrole group at 6.33 ppm to the styryl group at 6.83–7.17 ppm. The results varied from 76 to 86%, possibly due to the bimolecular termination enhanced by the high monomer conversion (>90% conversion).

**Figure 2:**
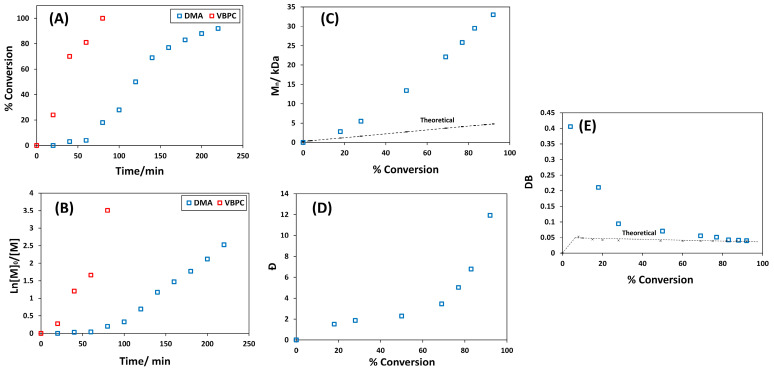
(**A**) Conversion vs. time plot. (**B**) Ln[M]_0_/[M] vs. time plot. (**C**) Dependence of *M_n_* on conversion in comparison with theoretical standard RAFT: *M_n,theo_* = [DMA]/[VBPC] × Mw of DMA × conversion + Mw of VBPC. (**D**) Dependence of *Ð* on DMA conversion. (**E**) *DB* vs. DMA conversion in comparison with theoretical values for RAFT copolymerization of DMA with VBPC at γ = 50 and 50 wt%.

In SCVP, similar reactivity of the vinyl group of the CTM with respect to the monomer is essential for the homogeneous distribution of the branch points and, thus, the formation of HBP. The formation of a hyperstar polymer is expected when the reactivity of the vinyl of CTM is much greater than the monomer, while a macroCTM is more likely to be formed in the opposite case [49,50]. In order to investigate the evolution of the structure, a moderate amount of the reaction mixture at γ = 50 and 50 wt% of DMA in dioxane was periodically sampled for ^1^H NMR analysis. The vinyl conversion of VBPC and DMA was calculated using the intensity change of the vinyl signals at 5.24 and 5.65 ppm, respectively. Despite its low concentration in the reaction mixture, the vinyl group of the VBPC was completely consumed within 80 min, accompanied by little DMA polymerization (Figure 2A). This difference in reactivity suggests that a homogeneous distribution of the branchpoints was not achieved and the polymer formed was not HBP but, more likely, a hyperstar polymer.

To further investigate the evolution of the polymer structure during polymerization, the dependence of *M_n_* and *Ð* on DMA conversion was studied. Ideally, when γ >> 1 and at low monomer conversion, *M_n_* increases linearly, while *Ð* decreases, as it should in standard RAFT polymerization. Then, both *M_n_* and *Ð* grow exponentially because of the nature of linking step-growth reactions [49,50]. However, this greatly depends on the reactivity of the CTM vinyl and the monomer. When the vinyl of CTM is less reactive than the monomer, *M_n_* grows almost linearly up to a very high conversion, similar to standard RAFT, with a relatively low *Ð* of less than 2, suggesting the production of linear macroCTM. When the vinyl of CTM is much more reactive than the monomer, *M_n_* and *Ð* strongly increase at a low monomer conversion compared with the standard RAFT, since CTM is consumed rapidly to form a hyperbranched core. As illustrated in Figure 2C, no peak of polymer or oligomer was noticed before 80 min (before 20% DMA conversion) despite the complete conversion of VBPC. At 20% conversion, a polymer with *M_n_* of 2.8 kDa and *Ð* of 1.8 was formed. This is much higher than the theoretical *M_n_* of 1.1 kDa for standard linear RAFT polymerization, indicating the formation of the hyperbranched core due to the high reactivity of the VBPC. Thereafter, both *M_n_* and *Ð* strongly grew throughout the polymerization.

The dependence of *DB* on the conversion of DMA was also investigated. In ideal SCVP and when γ ˃˃ 1, the *DB* should very quickly reach its final value and remain constant throughout the polymerization [49,50]. The dependence of *DB* on DMA conversion shown in Figure 2E provides sufficient evidence that the final structure is not HBP. The highest *DB* of 0.405 was achieved at a low DMA conversion of 4%, after which the *DB* began to decline. Considering the kinetic data for the two monomers, this possibly confirms that the VBPC underwent SCVP first to form a hyperbranched core and then grew DMA arms, forming a hyperstar polymer. Since star polymers have also been found to be good candidates for use as ^19^F MRI contrast agents [20,21,30], these polymers were used for the further synthesis of 5-FU polymer conjugates.

### 3.2. Synthesis of 5-FU Polymer Conjugate

**Figure 3:**
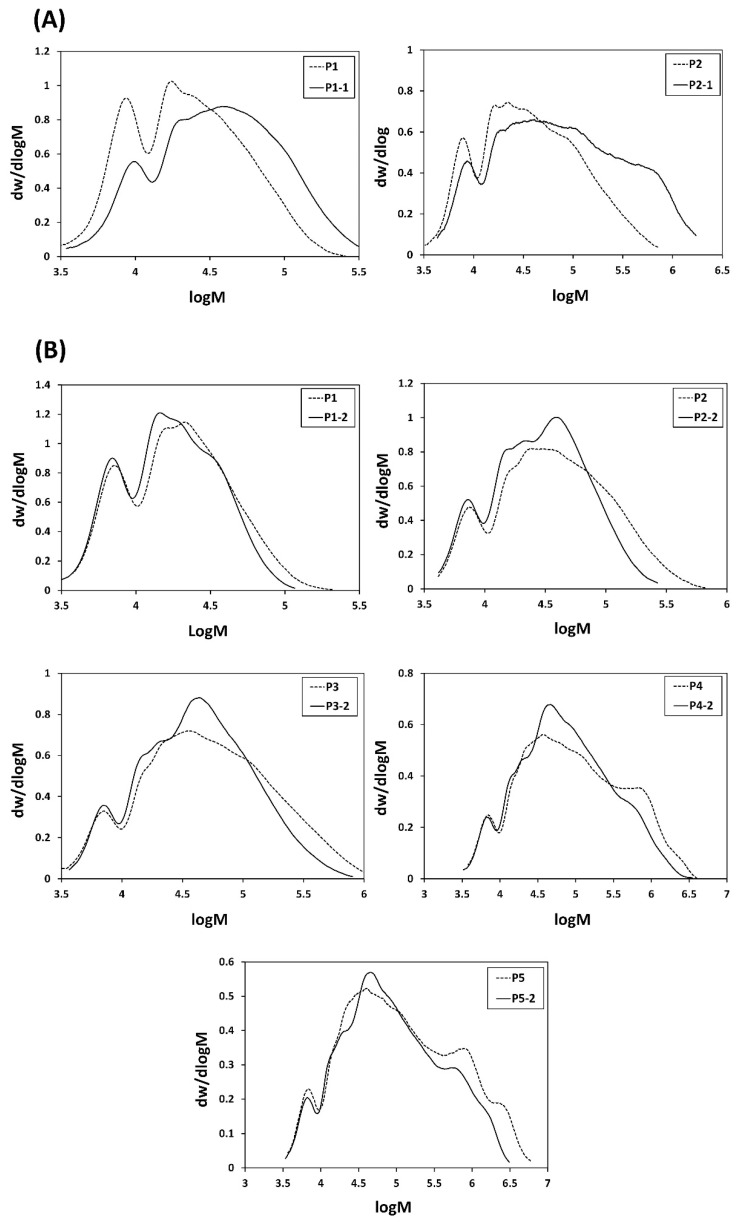
DMF GPC molecular weight distributions of HBPs before (dotted lines) and after aminolysis/HEA conjugation (plain lines) (**A**) in the absence Me_2_PPh, and (**B**) in the presence of Me_2_ PPh.

In situ aminolysis/Michael addition is one of the most widely used and versatile methods for the removal of the thiocarbonylthio end groups and to conjugate the desired moieties in a one-pot process [51,52]. Due to its multifunctional nature, however, care needs to be taken when removing RAFT end groups from HBPs, as incomplete capping with a Michael acceptor might lead to disulfide crosslinked materials. Therefore, the conjugation of the vinyl modified peptide into HBPs was first examined using a commercially available analogue, *N*-hydroxyethylacrylamide (HEA). The aminolysis of HBPs was conducted in the presence of excess propylamine and HEA in *N*,*N*-dimethyl formamide (DMF) (Scheme S1). The polymer was then purified through precipitation, followed by dialysis against deionized water. After the isolation of the polymers by precipitation, the high-molecular-weight polymers (i.e., P3, P4, and P5) completely turned to gel, probably due to the aerial oxidation of thiols into disulfide. This indicates that not all thiols were trapped within HEA. In contrast, no gel was formed after the modification of the low molecular weight polymers (i.e., P1 and P2). The GPC of these samples indicated the presence of high-molecular-weight disulfide contaminants (Figure 3A). In order to avoid the formation of disulfide, aminolysis was conducted in the presence of a reducing agent, phenyldimethylphosphine, Me_2_PPh, which also acts as a catalyst for subsequent Michael addition reactions [53,54,55,56,57]. When the polymers were modified in the presence of Me_2_PPh, no gel was formed, even for high-molecular-weight polymers. GPC of these samples indicates the absence of high-molecular-weight disulfide species, suggesting that all thiols were trapped with HEA (Figure 3B).

It is worth mentioning that our attempts to synthesize the desired hexa-peptide, Gly-Phe-Leu-Gly-Leu-Gly-α-(5-FU), using a standard solution peptide synthesis approach failed due to critical impurities and low yield. Accordingly, the assembly of the hexapeptide on HBP (as a water-soluble polymeric support) in a couple of steps was used as an alternative (Scheme 2B). Growing the peptide with the aid of water-soluble polymeric support allowed the use of an excess of reagents to drive the reaction to completion. Furthermore, excess amounts of reagents and by-products could be readily purified by membrane filtration. As shown in Scheme 2B, the vinyl-modified tetra-peptide was conjugated to P3 in the presence of Me_2_PPh to avoid the formation of disulfide contaminants. The change in color of P3 from yellow to white indicates the successful cleavage of thiocarbonylthio at the chain ends. UV absorbance of the thiocarbonylthio at around 300 nm was completely absent, indicating quantitative cleavage (Figure S7). ^1^H NMR in D_2_O confirmed the successful synthesis of the polymer–peptide conjugate (Figure S8). The disappearance of vinyl resonance signals between 5.50 and 6.50 ppm confirms the complete elimination of unreacted peptide by dialysis. Moreover, the presence of a new signal at 3.75 ppm, attributed to the formation of S-CH_2_ as a result of thiol addition to the vinyl-modified peptide, confirms that conjugation was successful. Considering that the tetra-peptide is water insoluble, the observation of resonance signals characteristic of the peptide in D_2_O can only be possible after the conjugation to the water-soluble polymer.

The 5-FU prodrug (Leu-Gly(5-FU)) was finally synthesized following the previously reported procedure [41], with some modifications. This included the optimization of benzyloxycarbonyl group (Cbz) cleavage using catalytic hydrogenation in the presence of various hydrogen transfer agents (Table S3). Among the various hydrogen transfer agents used, Cbz cleavage was only achieved in the presence of formic acid (100% cleavage) or 1,4-cyclohexadiene (79% cleavage). Despite achieving quantitative cleavage of Cbz when formic acid is used, partial hydrogenation of the double bond in the pyrimidine ring of 5-FU was observed (Figure S9). In contrast, no side product was formed when 1,4-cyclohexadiene was used. The 5-FU prodrug was then attached to the polymer according to the procedure illustrated in Scheme 2B. ^19^F NMR of the intermediate (Figure S10), along with ^19^F NMR and ^1^H NMR of the final product, indicate the formation of the dipeptide and the desired hexapeptide conjugates (Figure S11). HBP can originally have carboxylic end groups, produced by bimolecular termination with ACVA radicals, which might form the dipeptide conjugate.

### 3.3. Monitoring 5-FU Release Using ^19^F NMR

The ability to monitor 5-FU release using ^19^F NMR for MRI applications was then examined. The degradation mechanism of 5-FU polymer conjugate in the presence of tritosomes (mixture of lysosomal enzymes well known to be highly expressed in cancer cells) involves the release of a dipeptide derivative of 5-FU, which further degrades into free 5-FU [41,58]. However, the degradation mechanism might vary depending on the enzymes used [59]. To avoid handling animals, the release of 5-FU was studied using commercial S9 fraction from rat liver instead. The S9 fraction is a mixture of microsomes and cytosol and contains a wide range of metabolizing enzymes [60].

**Figure 4:**
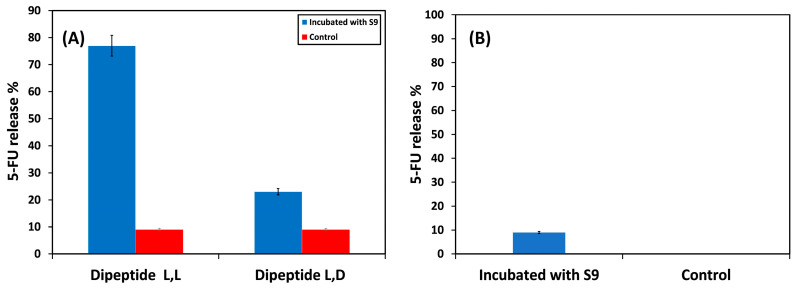
Incubation of (**A**) dipeptide derivatives of 5-FU and (**B**) 5-FU polymer conjugate with the liver S9 fraction at 37 °C pH 7.4 for 24 h.

The release of 5-FU from L,L and L,D dipeptides was initially investigated in an NMR tube by incubating 15 µM 5-FU with liver S9 fractions in 1:9 D_2_O:PBS containing 3 mM MgCl_2_ (pH 7.4) at 37 °C for 24 h. The release was monitored using 1D ^19^F NMR, along with ^19^F *T*_1_/*T*_2_. As illustrated in Figure 4A, upon incubation of the dipeptides with S9, the release of 5-FU from the L,L dipeptide was almost five times the 5-FU released from the L,D dipeptide. The incubation of L,L dipeptide with S9 fractions resulted in quantitative degradation (almost 77% release of 5-FU) whereas the incubation of L,D dipeptide resulted in only 15% 5-FU cleavage. This result is in agreement with the results reported by Putnam et al. [41,58]. This is not a surprising result since amino acids found in the body often have a levorotatory configuration, and the enzymes can have very specific stereoselectivity within their active sites.

^19^F NMR spectra before and after incubation of L,L dipeptide with liver S9 fractions are shown in Figure S13. Upon incubation with the enzyme, the disappearance of the broad singlet due to the dipeptide at −166.93 ppm was recorded, accompanied by the presence of a new sharp doublet signal due to free 5-FU at −169.16 ppm. This suggests that the ^19^F nuclei of the dipeptide experiences a slow tumbling rate in comparison to the free 5-FU. The slow tumbling rate was clearly verified by ^19^F spin–lattice *T*_1_ and spin–spin *T*_2_ relaxation times of the incubated sample determined by standard inversion–recovery and Carr–Purcell–Meiboom–Gill pulse sequences, respectively. As anticipated, *T*_1_ and *T*_2_ relaxation times of the incubated sample were considerably elongated from 0.997 to 4.804 s and from 0.047 to 0.144 s, respectively. However, the *T*_2_ relaxation time of the released 5-FU is still lower than the original value of 0.476 s for free 5-FU (Table S4), most likely because its mobility was hindered by the S9 fraction. The ^19^F NMR spectrum also displays two other signals at −166.42 and −166.10 ppm (+2.74 and 3.06 ppm relative to 5-FU), which are probably due to metabolites of 5-FU. According to the pH titration curves of 5-FU metabolites studied by Lutz and Hull [61], the two signals are perhaps assigned to 5-fluouridine and 5-fluoro-2-deoxyuridine, respectively.

Since the enzyme is stereoselective, only the polymer conjugate with the L,L configuration was made and examined. The incubation of the polymer conjugate with the S9 fraction resulted in a very low (9%) cleavage of 5-FU (Figure 4B) in comparison with the quantitative cleavage reported by rat liver tritosomes at pH 5.5 of 5-FU and 5-FU derivatives [41,58]. The low percent release can be due to the limited accessibility of the S9 fraction to the oligo-peptide spacer due to the relatively compact structure within the polymer conjugate.

**Figure 5:**
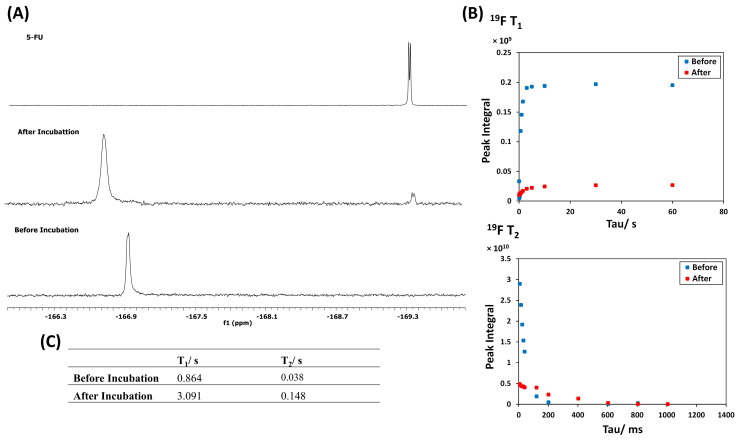
(**A**) ^19^F NMR spectrum of 5-FU polymer conjugate in PBS/D_2_O (9:1) containing 3 mM MgCl_2_ before and after the incubation with the S9 fraction from the liver. (**B**) ^19^F *T*_1_ and *T*_2_ relaxation time curves of the polymer and the released 5-FU. (**C**) List of ^19^F *T*_1_ and *T*_2_ relaxation times of the polymer and the released 5-FU.

^19^F NMR spectra of the polymer conjugate before and after the incubation with the liver S9 fraction are shown in Figure 5. After incubation with S9, a sharp doublet resonance at −169.19 ppm due to the cleaved 5-FU was observed. As expected, the *T*_1_ and *T*_2_ ^19^F relaxation times of the free drug showed a significant increase from 0.846 to 3.091 s and from 0.038 to 0.148 s, respectively, indicating an enhancement of the tumbling rate of the ^19^F nuclei. As the MRI signal intensity is directly proportional to the *T*_2_ relaxation time, a switch of the signal to ON is expected upon 5-FU release. Both the *T*_1_ and *T*_2_ relaxation times of the released drug are still less than the original values of the free 5-FU illustrated in Table S4 (4.866 s for *T*_1_ and 0.476 s for *T*_2_). This is possibly because the mobility of the cleaved 5-FU was hindered by the enzyme. Furthermore, no resonance signal due to the dipeptide was observed in the ^19^F NMR spectrum, indicating that all the dipeptide converted to 5-FU, which is in a good agreement with the result of the incubation of the L,L dipeptide alone with the S9 fractions (Figure S12). This change in the *T*_2_ relaxation time highlights the success with which ^19^F MRI may be used to monitor the release of the fluorinated drug from a macromolecule drug delivery vehicle upon incubation with a cleaving enzyme. While these results suggest that this approach is valid, further optimization of conditions to enhance the drug release is required.

## 4. Conclusions

In conclusion, a new ^19^F MRI contrast agent based upon HBP conjugation with an oligopeptide bearing 5-FU for monitoring drug release was developed and examined. ^19^F nuclei within the 5-FU polymer conjugate and the free 5-FU had significantly different mobilities due to their different molecular sizes. This was measured through ^19^F *T*_2_ relaxation times. Upon incubation with the S9 fraction, free 5-FU was released, accompanied by a significant increase in its *T*_2_ relaxation times from 0.038 to 0.128 s. However, the S9 fraction revealed a low efficiency of only 9% 5-FU release. Therefore, 5-FU release could not be further evaluated using ^19^F MRI phantom imaging. 5-FU release must be maximized to enhance the signal-to-noise ratio, as the fluorine concentration is directly proportional to ^19^F MRI signal intensity. In principle, this approach is not limited to monitoring 5-FU release but could be applicable to monitoring the release of other fluorinated drugs and using linkers responsive to other types of stimuli such as pH and redox. This can also be expanded to study the drug release from stimuli-responsive encapsulated systems.

## Figures and Tables

**Table 1:** Results of RAFT-SCVP copolymerization of DMA with VBPC in dioxane at 60 °C at γ = 50.

Polymer	Γ	Time/h	Conv/% ^a^	*M_n_*/kDa ^b^	*Ð* ^b^	*M_n,theo_*/kDa ^c^	*DB*_(exp)_ ^d^	*DB*_theo_ ^e^	F ^f^
P1	50	24	91%	13.5	1.78	4.8	0.038	0.039	67
P2	50	24	96%	19.9	2.70	5.0	0.038	0.039	76
P3	50	7	93%	25.5	3.77	4.9	0.038	0.039	75
P4	50	6	91%	32.0	8.11	4.8	0.038	0.039	72
P5	50	4	92%	33.0	11.92	4.9	0.038	0.039	73

^a^ Determined by ^1^H NMR spectroscopy of the crude reaction mixture, ^b^ Measured by GPC in DMF calibrated with linear PMMA homopolymer standards, ^c^ Calculated considering the formula of the linear RAFT polymerization M_n,theo_ = [DMA]/[VBPC] × M_w_ of DMA × conversion + M_w_ of VBPC, ^d^ Calculated using Equation (3), ^e^ Calculated using *DB*_theo_ = ((2(1−e^(−(γ + 1) xm^))[x_m_ − [(1−e ^(−(γ + 1) xm)^)/(γ + 1)]])/(γx_m_ + 1 − (1 − x_m_)(2 − e^−(γ + 1) xm^)) where γ and x_m_ refer to [DMA]:[VBPC] ratio and DMA conversion, respectively, ^f^ The fidelity of the thiocarbonylthio end groups is calculated by comparing the integration of pyrrole protons at 6.43 ppm to styryl protons at 7.05 ppm.

## Data Availability

Not applicable.

## Supplementary Materials

The following supporting information can be downloaded at: https://www.mdpi.com/article/10.3390/polym15071778/s1, Figure S1. ^1^H NMR of hyper-branched poly(*N*,*N*-dimethylacrylamide) (HB-PDMA) and linear poly(*N*,*N*-dimethylacrylamide) (L-PDMA) in CDCl_3_: Figure S2. Molecular weight distributions of HBPs at γ= 20, 30 & 40 determined by DMF GPC relative to poly (methyl methacrylate) (PMMA) standards: Figure S3. (A) Conversion vs time plot, (B) Ln[M]_0_/[M] vs time plot of RAFT copolymerization of DMA with VBPC at γ = 50 and concentration from 10wt% to 50wt%: Figure S4. (A) Dependence of *M_n_* on conversion, (B) Dependence of *Ð* on DMA conversion, for RAFT copolymerization of DMA with VBPC at γ = 50 and 50 wt%: Figure S5. ^1^H NMR of non-modified and vinyl modified peptide in (CD_3_)_2_SO: Figure S6. HPLC profiles of (A) non-modified peptide 3 (B) vinyl-modified peptide. Column: Waters XBridge C18 250 × 4.6 mm. Mobile phase: gradient 5 to 95% acetonitrile (with 0.1% formic acid) over 20 min at a flow rate of 1 mL min^−1^: Figure S7. UV-vis absorbance spectra of HB-PDMA before and after aminolysis in water: Figure S8. ^1^H NMR spectra of HB-PDMA-Gly-Leu-Phe-Gly in D_2_O and vinyl-modified tetra-peptide (Gly-Leu-Phe-Leu) in (CD_3_)_2_SO: Figure S9. ^1^H NMR spectrum in (CD_3_)_2_SO of the isolated side product after the hydrogenation of 5-FU prodrug with formic acid at room temperature. The disappearance of the doublet at 8.16 ppm due to pyrimidine CH proton along with the presence of the resonance signals for CH_2_-CHF protons (3.86 and 5.12–5.26 ppm) indicates the hydrogenation of the double bond in the pyrimidine ring: Figure S10. ^19^F NMR of HB-PDMA-Gly-Leu-Phe-Gly with pentafluorophenyl ester end group in (CD_3_)_2_SO: Figure S11. ^1^H NMR and ^19^F NMR of HB-PDMA-Gly-Leu-Phe-Gly-Leu-Gly(5-FU) in D_2_O. The two signals due to the α-hydrogen of leucine at 4.21 and 4.38 ppm indicates different environments due to the formation of the dipeptide and the desired hexapeptide conjugates: Figure S12. (A) Size distribution of the polymer peptide conjugate at pH 7.4 determined by DLS (B) Autocorrelation curve: Figure S13. (A) ^19^F NMR spectrum of L,L dipeptide before and after the treatment with S9 fraction from liver in PBS:D_2_O 9:1 containing 3 mM MgCl_2_ (B) ^19^F *T*_1_ and *T*_2_ relaxation times curves of L,L dipeptide before and after treatment (C) list of ^19^F *T*_1_ and *T*_2_ relaxation times of L,L dipeptide before and after the treatment: Table S1. Results of RAFT-SCVP copolymerization of DMA with VBPC in dioxane at 60 °C at different feed ratios γ =20–40: Table S2. A comparison between DLS and TEM data for HBPs: Table S3. Cbz deprotection using various hydrogen transfer agents: Table S4. List of ^19^F *T*_1_ and *T*_2_ relaxation times of 5-FU, 5-FU prodrug, and the polymer conjugate at pH 7.4: Scheme S1. One-pot aminolysis/ N-hydroxyethylacrylamide conjugation

## References

[1] Lauterbur P.C. (1973). Image Formation by Induced Local Interactions: Examples Employing Nuclear Magnetic Resonance. Nature.

[2] James M.L., Gambhir S.S. (2012). A Molecular Imaging Primer: Modalities, Imaging Agents, and Applications. Physiol. Rev..

[3] Debbage P., Jaschke W. (2008). Molecular Imaging with Nanoparticles: Giant Roles for Dwarf Actors. Histochem. Cell Biol..

[4] Aime S., Botta M., Terreno E. (2005). Gd(III)-Based Contrast Agents for MRI. Adv. Inorg. Chem..

[5] Caravan P., Ellison J.J., McMurry T.J., Lauffer R.B. (1999). Gadolinium(III) Chelates as MRI Contrast Agents: Structure, Dynamics, and Applications. Chem. Rev..

[6] Bulte J.W.M., Kraitchman D.L. (2004). Iron Oxide MR Contrast Agents for Molecular and Cellular Imaging. NMR Biomed..

[7] Laurent S., Forge D., Port M., Roch A., Robic C., Elst L.V., Muller R.N. (2008). Magnetic Iron Oxide Nanoparticles: Synthesis, Stabilization, Vectorization, Physicochemical Characterizations, and Biological Applications. Chem. Rev..

[8] Peng X.H., Qian X., Mao H., Wang A.Y., Chen Z.G., Nie S., Shin D.M. (2008). Targeted Magnetic Iron Oxide Nanoparticles for Tumor Imaging and Therapy. Int. J. Nanomed..

[9] Code R.F., Harrison J.E., Mcneill K.G., Szyjkowski M. (1990). In Vivo ^19^F Spin Relaxation in Index Finger Bones. Magn. Reson. Med..

[10] Bovey F.A. (1988). Nuclear Magnetic Resonance Spectroscopy.

[11] Bober Z., Aebisher D., Ożóg Ł., Tabarkiewicz J., Tutka P., Bartusik-Aebisher D. (2017). ^19^F MRI As a Tool for Imaging Drug Delivery to Tissue and Individual Cells. Eur. J. Clin. Exp. Med..

[12] Zhao D., Constantinescu A., Jiang L., Hahn E.W., Mason R.P. (2001). Prognostic Radiology: Quantitative Assessment of Tumor Oxygen Dynamics by MRI. Am. J. Clin. Oncol. Cancer Clin. Trials.

[13] Srinivas M., Heerschap A., Ahrens E.T., Figdor C.G., de Vries I.J.M. (2010). ^19^F MRI for Quantitative in Vivo Cell Tracking. Trends Biotechnol..

[14] Srinivas M., Boehm-Sturm P., Figdor C.G., de Vries I.J., Hoehn M. (2012). Labeling Cells for in Vivo Tracking using ^19^F MRI. Biomaterials.

[15] Ahrens E.T., Zhong J. (2013). In Vivo MRI Cell Tracking Using Perfluorocarbon Probes and Fluorine-19 Detection. NMR Biomed..

[16] Knight J.C., Edwards P.G., Paisey S.J. (2011). Fluorinated Contrast Agents for Magnetic Resonance Imaging; a Review of Recent Developments. RSC Adv..

[17] Tirotta I., Dichiarante V., Pigliacelli C., Cavallo G., Terraneo G., Bombelli F.B., Metrangolo P., Resnati G. (2015). ^19^F Magnetic Resonance Imaging (MRI): From Design of Materials to Clinical Applications. Chem. Rev..

[18] Srinivas M., Morel P.A., Ernst L.A., Laidlaw D.H., Ahrens E.T. (2007). Fluorine-19 MRI for Visualization and Quantification of Cell Migration in a Diabetes Model. Magn. Reson. Med..

[19] Zhang C., Moonshi S.S., Han Y., Puttick S., Peng H., Magoling B.J.A., Reid J.C., Bernardi S., Searles D.J., Král P. (2017). PFPE-Based Polymeric ^19^F MRI Agents: A New Class of Contrast Agents with Outstanding Sensitivity. Macromolecules.

[20] Wang K., Peng H., Thurecht K.J., Puttick S., Whittaker A.K. (2013). pH-Responsive Star Polymer Nanoparticles: Potential ^19^F MRI Contrast Agents for Tumour-Selective Imaging. Polym. Chem..

[21] Wang K., Peng H., Thurecht K.J., Whittaker A.K. (2016). Fluorinated POSS-Star Polymers for ^19^F MRI. Macromol. Chem. Phys..

[22] Du W.J., Nystrom A.M., Zhang L., Powell K.T., Li Y.L., Cheng C., Wickline S.A., Wooley K.L. (2008). Amphiphilic Hyperbranched Fluoropolymers as Nanoscopic F-19 Magnetic Resonance Imaging Agent Assemblies. Biomacromolecules.

[23] Thurecht K.J., Blakey I., Peng H., Squires O., Hsu S., Alexander C., Whittaker A.K. (2010). Functional Hyperbranched Polymers: Toward Targeted in Vivo ^19^F Magnetic Resonance Imaging Using Designed Macromolecules. J. Am. Chem. Soc..

[24] Oishi M., Sumitani S., Nagasaki Y. (2007). On-Off Regulation of ^19^F Magnetic Resonance Signals Based on pH-Sensitive PEGylated Nanogels for Potential Tumor-Specific Smart ^19^F MRI Probes. Bioconjug. Chem..

[25] Bailey M.M., Mahoney C.M., Dempah K.E., Davis J.M., Becker M.L., Khondee S., Munson E.J., Berkland C. (2010). Fluorinated Copolymer Nanoparticles for Multimodal Imaging Applications. Macromol. Rapid Commun..

[26] Wang K., Peng H., Thurecht K.J., Puttick S., Whittaker A.K. (2015). Segmented Highly Branched Copolymers: Rationally Designed Macromolecules for Improved and Tunable ^19^F MRI. Biomacromolecules.

[27] Wang K., Peng H., Thurecht K.J., Puttick S., Whittaker A.K. (2016). Multifunctional Hyperbranched Polymers for CT/19 F MRI Bimodal Molecular Imaging. Polym. Chem..

[28] Rolfe B.E., Blakey I., Squires O., Peng H., Boase N.R.B., Alexander C., Parsons P.G., Boyle G.M., Whittaker A.K., Thurecht K.J. (2014). Multimodal Polymer Nanoparticles with Combined ^19^F Magnetic Resonance and Optical Detection for Tunable, Targeted, Multimodal Imaging in Vivo. J. Am. Chem. Soc..

[29] Zhang C., Moonshi S.S., Wang W., Ta H.T., Han Y., Han F.Y., Peng H., Kra P., Rolfe B.E., Gooding J.J. (2018). High F-Content Perfluoropolyether-Based Nanoparticles for Targeted Detection of Breast Cancer by ^19^F Magnetic Resonance and Optical Imaging. ACS Nano.

[30] Wang K., Peng H., Thurecht K.J., Puttick S., Whittaker A.K. (2014). Biodegradable Core Crosslinked Star Polymer Nanoparticles as ^19^F MRI Contrast Agents for Selective Imaging. Polym. Chem..

[31] Fuchs A.V., Bapat A.P., Cowin G.J., Thurecht K.J. (2017). Switchable ^19^F MRI Polymer Theranostics: Towards in Situ Quantifiable Drug Release. Polym. Chem..

[32] Fu C., Herbst S., Zhang C., Whittaker A.K. (2017). Polymeric ^19^F MRI Agents Responsive to Reactive Oxygen Species. Polym. Chem..

[33] Mizukami S., Takikawa R., Sugihara F., Shirakawa M., Kikuchi K. (2009). Dual-Function Probe to Detect Protease Activity for Fluorescence Measurement and ^19^F MRI. Angew. Chem. Int. Ed..

[34] Keliris A., Mamedov I., Hagberg G.E., Logothetis N.K., Scheffler K., Engelmann J. (2012). A smart^19^F and ^1^H MRI Probe with Self-Immolative Linker as a Versatile Tool for Detection of Enzymes. Contrast Media Mol. Imaging.

[35] Mizukami S., Matsushita H., Takikawa R., Sugihara F., Shirakawa M., Kikuchi K. (2011). ^19^F MRI Detection of β-Galactosidase Activity for Imaging of Gene Expression. Chem. Sci..

[36] Mizukami S., Takikawa R., Sugihara F., Hori Y., Tochio H., Wälchli M., Shirakawa M., Kikuchi K. (2008). Paramagnetic Relaxation-Based ^19^F MRI Probe to Detect Protease Activity. J. Am. Chem. Soc..

[37] Takaoka Y., Sakamoto T., Tsukiji S., Narazaki M., Matsuda T., Tochio H., Shirakawa M., Hamachi I. (2009). Self-Assembling Nanoprobes That Display Off/on ^19^F Nuclear Magnetic Resonance Signals for Protein Detection and Imaging. Nat. Chem..

[38] Yu J., Otten P., Ma Z., Cui W., Liu L., Mason R.P. (2004). Novel NMR Platform for Detecting Gene Transfection: Synthesis and Evaluation of Fluorinated Phenyl β-D-Galactosides with Potential Application for Assessing lacZ Gene Expression. Bioconjug. Chem..

[39] Kodibagkar V.D., Yu J., Liu L., Hetherington H.P., Mason R.P. (2006). Imaging β-Galactosidase Activity using ^19^F Chemical Shift Imaging of LacZ Gene-Reporter Molecule 2-Fluoro-4-Nitrophenol-β-D-Galactopyranoside. Magn. Reson. Imaging.

[40] Liu L., Kodibagkar V.D., Yu J.-X., Mason R.P. (2007). ^19^F-NMR Detection of lacZ Gene Expression via the Enzymic Hydrolysis of 2-Fluoro-4-Nitrophenyl -D-Galactopyranoside in Vivo in PC3 Prostate Tumor Xenografts in the Mouse. FASEB J..

[41] Putnam D., Kopeček J. (1995). Enantioselective Release of 5-Fluorouracil from N-(2-Hydroxypropyl)Methacrylamide-Based Copolymers via Lysosomal Enzymes. Bioconjug. Chem..

[42] Plenderleith R., Swift T., Rimmer S. (2014). Highly-Branched poly(N-Isopropyl Acrylamide)s with Core–shell Morphology below the Lower Critical Solution Temperature. RSC Adv..

[43] Coin I., Beyermann M., Bienert M. (2007). Solid-Phase Peptide Synthesis: From Standard Procedures to the Synthesis of Difficult Sequences. Nat. Protoc..

[44] Nkhifor M., Schacht E.H. (1994). Synthesis of Peptide Derivatives of Hluorouracil. Tetrahedron.

[45] Frechet J.M.J., Henmi M., Gitsov I., Aoshima S., Leduc M.R., Grubbs R.B. (1995). Self-Condensing Vinyl Polymerization—An Approach to Dendritic Materials. Science.

[46] Rimmer S., Carter S., Rutkaite R., Haycock J.W., Swanson L. (2007). Highly Branched Poly-(N-Isopropylacrylamide)s with Arginine–glycine–aspartic Acid (RGD)- or COOH-Chain Ends That Form Sub-Micron Stimulus-Responsive Particles above the Critical Solution Temperature. Soft Matter.

[47] Platt L., Kelly L., Rimmer S. (2014). Controlled Delivery of Cytokine Growth Factors Mediated by Core–shell Particles with Poly(acrylamidomethylpropane Sulphonate) Shells. J. Mater. Chem. B.

[48] Shallcross L., Roche K., Wilcock C.J., Stanton K.T., Swift T., Rimmer S., Hatton P.V., Spain S.G. (2017). The Effect of Hyperbranched Poly(acrylic Acid)s on the Morphology and Size of Precipitated Nanoscale (Fluor)hydroxyapatite. J. Mater. Chem. B.

[49] Litvinenko G.I., Simon P.F.W., Müller A.H.E. (1999). Molecular Parameters of Hyperbranched Copolymers Obtained by Self-Condensing Vinyl Copolymerization. 1. Equal Rate Constants. Macromolecules.

[50] Litvinenko G.I., Simon P.F.W., Müller A.H.E. (2001). Molecular Parameters of Hyperbranched Copolymers Obtained by Self-Condensing Vinyl Copolymerization, 2. Non-Equal Rate Constants. Macromolecules.

[51] Qiu X.P., Winnik F.M. (2006). Facile and Efficient One-Pot Transformation of RAFT Polymer End Groups via a Mild Aminolysis/michael Addition Sequence. Macromol. Rapid Commun..

[52] Grover G.N., Alconcel S.N.S., Matsumoto N.M., Maynard H.D. (2009). Trapping of Thiol-Terminated Acrylate Polymers with Divinyl Sulfone to Generate Well-Defined Semitelechelic Michael Acceptor Polymers. Macromolecules.

[53] Chan J.W., Yu B., Hoyle C.E., Lowe A.B. (2008). Convergent Synthesis of 3-Arm Star Polymers from RAFT-Prepared poly(N,N-Diethylacrylamide) via a Thiol–ene Click Reaction. Chem. Commun..

[54] Yu B., Chan J.W., Hoyle C.E., Lowe A.B. (2009). Sequential Thiol-Ene/Thiol-Ene and Thiol-Ene/Thiol-Yne Reactions as a Route to Well-Defined Mono and Bis End-Functionalized Poly(N-Isopropylacrylamide). J. Polym. Sci. Part A Polym. Chem..

[55] Boyer C., Granville A., Davis T.P., Bulmus V. (2009). Modification of RAFT-Polymers via Thiol-Ene Reactions: A General Route to Functional Polymers and New Architectures. J. Polym. Sci. Part A Polym. Chem..

[56] Li G.-Z., Randev R.K., Soeriyadi A.H., Rees G., Boyer C., Tong Z., Davis T.P., Becer C.R., Haddleton D.M. (2010). Investigation into Thiol-(Meth)acrylate Michael Addition Reactions Using Amine and Phosphine Catalysts. Polym. Chem..

[57] Chan J.W., Yu B., Hoyle C.E., Lowe A.B. (2009). The Nucleophilic, Phosphine-Catalyzed Thiol-Ene Click Reaction and Convergent Star Synthesis with RAFT-Prepared Homopolymers. Polymers.

[58] Putnam D.A., Shiah J.G., Kopeček J. (1996). Intracellularly Biorecognizable Derivatives of 5-Fluorouracil. Implications for Site-Specific Delivery in the Human Condition. Biochem. Pharmacol..

[59] Nichifor M., Schacht E.H., Seymour L.W. (1997). Polymeric Prodrugs of 5-Fluorouracil. J. Control. Release.

[60] Taylor J.B., Triggle D.J. (2007). Comprehensive Medicinal Chemistry II.

[61] Lutz N.W., Hull W.E. (1999). Assignment and pH Dependence of the ^19^F-NMR Resonances from the Fluorouracil Anabolites Involved in Fluoropyrimidine Chemotherapy. NMR Biomed..

