# Supplementary material for: Synthesis of 5-Fluorouracil Polymer Conjugate and 19F NMR Analysis of Drug Release for MRI Monitoring
Source: Polymers (Basel). 2023 Apr 3;15(7):1778. doi: 10.3390/polym15071778 (PMC10097235; doi:10.3390/polym15071778)
Supplement: Supplementary file 1 [file polymers-15-01778-s001.zip › polymers-2157264-supplementary.pdf]

Electronic Supporting Information for:

Synthesis of 5-Fluoruracil Polymer Conjugate and ^{19}F NMR Analysis of Drug Release for MRI Monitoring

Laila M. Alhaidari [1] and Sebastian G. Spain [2,*]

[1] Department of Chemistry, Faculty of Science, University of Majmaah, Majmaah, 11952, Saudi Arabia; l.alhaidari@mu.edu.sa
[2] Department of Chemistry, Dainton Building; University of Sheffield, Sheffield, S3 7HF, UK; s.g.spain@sheffield.ac.uk
***** Correspondence: s.g.spain@sheffield.ac.uk

Contents:

1 Synthesis of hyper-branched poly(*N*,*N*-dimethylacrylamide) (HB-PDMA) 2

1.1 NMR of hyper-branched poly(N,N-dimethylacrylamide) (HB-PDMA) 2

1.2 HB-PDMA with various molar ratio γ 2

1.3 HB-PDMA with various concentration and constant molar ratio γ=50 3

2 Synthesis of 5-FU polymer conjugate 4

2.1 Aminolysis in the presence of *N*-hydroxyethylacrylamide (HEA) as a Michael acceptor 4

2.2 The synthesis and attachment of vinyl-modified tetra-peptide (Gly-Leu-Phe-Gly) 5

2.2.1 The synthesis of vinyl-modified tetra-peptide (Gly-Leu-Phe-Gly) 5

2.2.2 The conjugation of vinyl-modified tetra-peptide (Gly-Leu-Phe-Gly) into HB-PDMA 6

2.3 The synthesis and attachment of the dipeptide, Leu-Gly(5-FU) 7

2.3.1 The synthesis of the dipeptide, Leu-Gly(5-FU) 7

2.3.2 The conjugation of the dipeptide, Leu-Gly(5-FU) 8

3 Monitoring the release of 5-FU using ^{19}F NMR 9

1 Synthesis of hyper-branched poly(*N*,*N*-dimethylacrylamide) (HB-PDMA)

1.1 NMR of hyper-branched poly(*N*,*N*-dimethylacrylamide) (HB-PDMA)

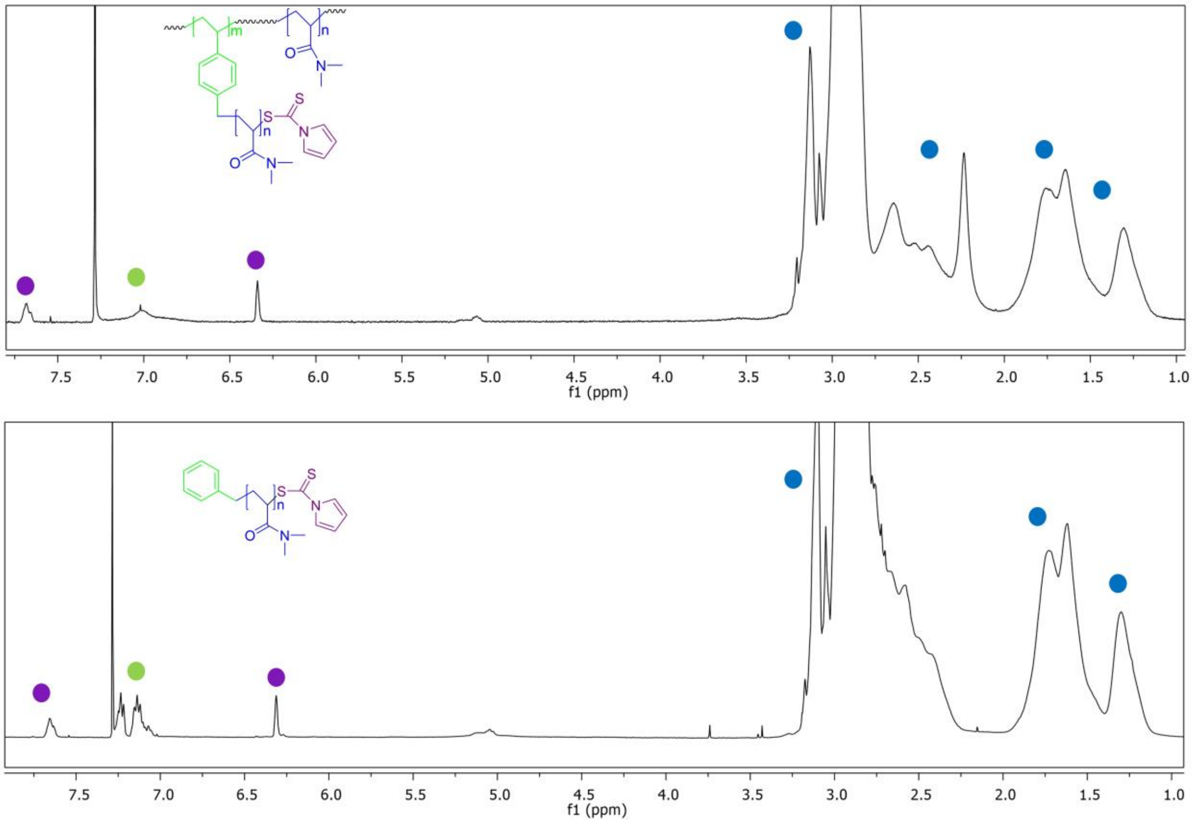

Figure S1.^{1}H NMR of hyper-branched poly(*N*,*N*-dimethylacrylamide) (HB-PDMA) and linear poly(*N*,*N*-dimethylacrylamide) (L-PDMA) in $CDCl_3$

1.2 HB-PDMA with various molar ratio γ

Table S1. Results of RAFT-SCVP copolymerization of DMA with VBPC in dioxane at 60 °C at different feed ratios γ =20-40

γ	Time / h	Conv / % [a]	M_n / kDa[b]	M_w / kDa[b]	Đ[b]	DB[c]	DB[d]
**20**	24	99	7.5	12	1.6	0.088	0.095
**30**	24	99	10	16	1.7	0.062	0.064
**40**	24	99	11	25	2.2	0.052	0.048

[a] Calculated by ^{1}H NMR spectroscopy of crude reaction mixture, [b] Measured by GPC in DMF calibrated with linear PMMA homopolymer standards, [c] calculated degree of branching, [d] theoretical degree of branching

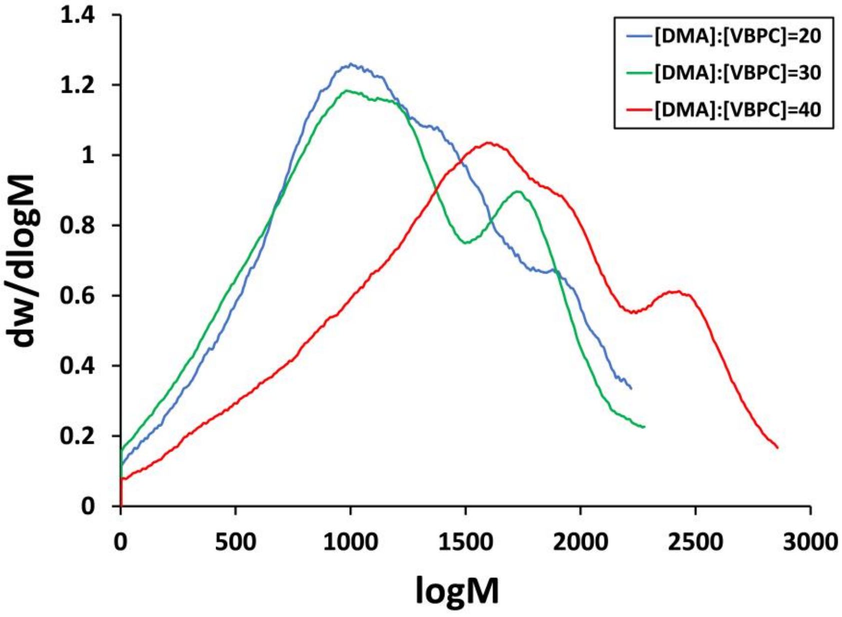

Figure S2. Molecular weight distributions of HBPs at γ= 20, 30 & 40 determined by DMF GPC relative to poly (methyl methacrylate) (PMMA) standards

1.3 HBPs with various concentration and constant molar ratio γ=50

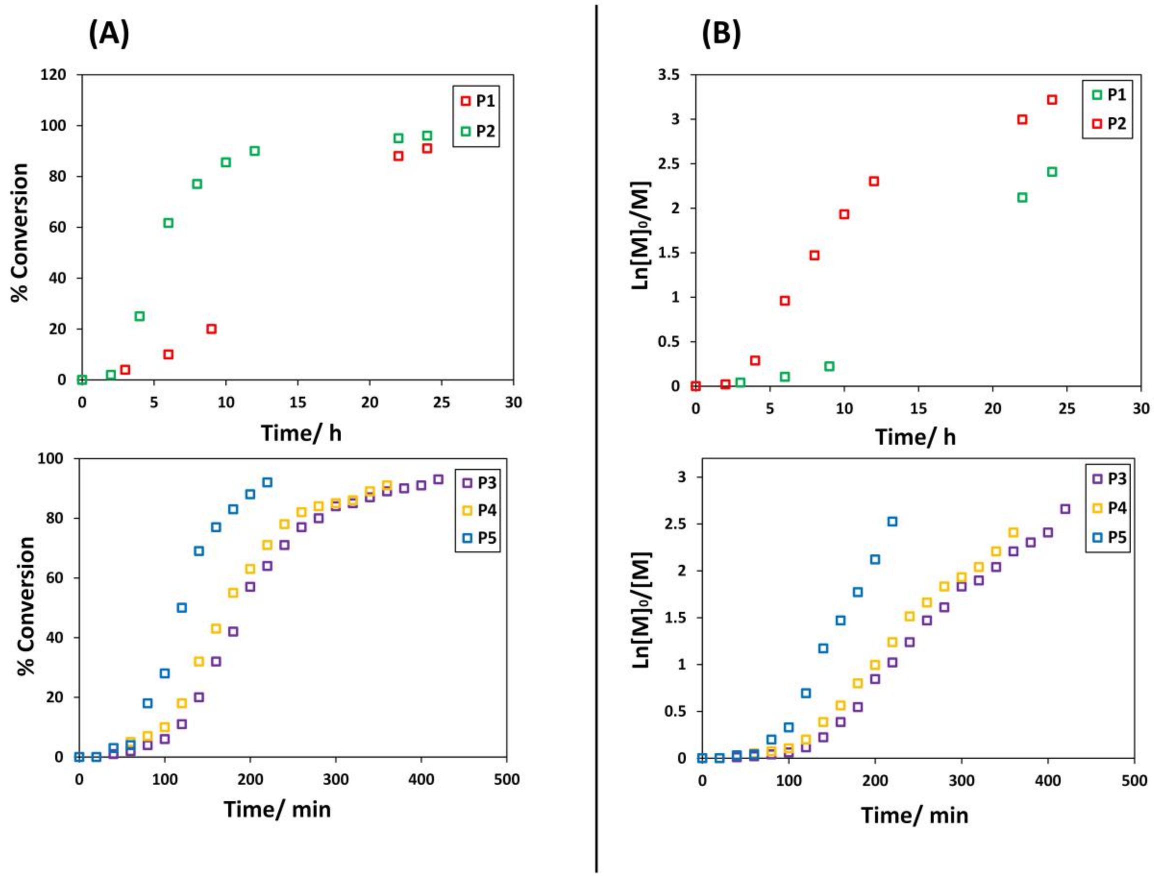

Figure S3. (A) Conversion vs time plot, **(B)** $Ln[M]_0/[M]$ vs time plot of RAFT copolymerization of DMA with VBPC at γ = 50 and concentration from 10wt% to 50wt%.

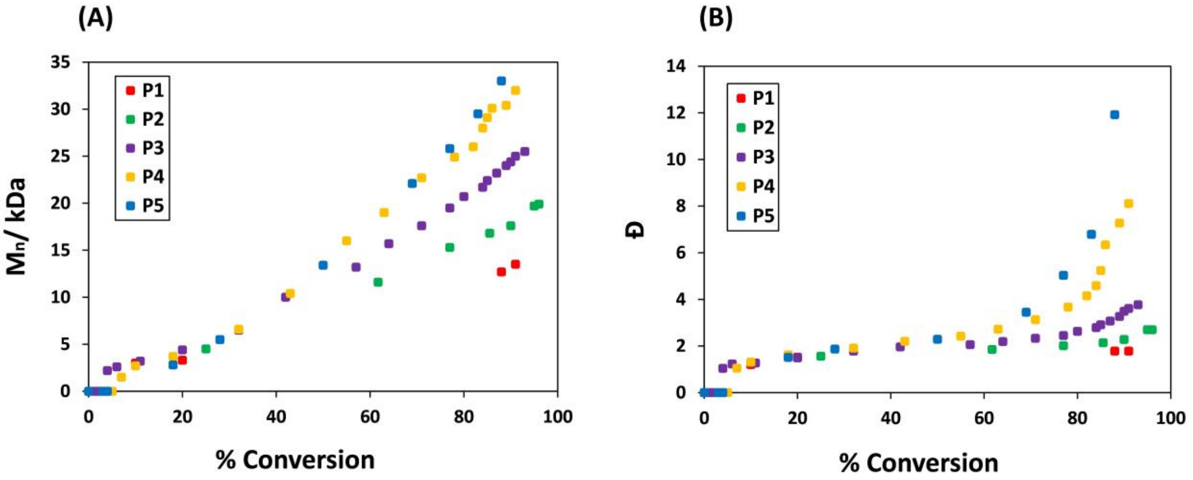

Figure S4. (A) Dependence of M_n on conversion (B) Dependence of Đ on DMA conversion, for RAFT copolymerization of DMA with VBPC at γ = 50 and 10-50 wt%

Table S2. A comparison between DLS and TEM data for HBPs

HBP	DLS		TEM	
	1st distribution/ nm	2nd distribution/ nm	1st distribution/ nm	2nd distribution/ nm
**P1**	7.5	76.1	3.5	15.0
**P2**	16.7	195.7	3.0	24.0
**P3**	49.9	-	-	
**P4**	82.2	-	-	
**P5**	24.1	125.7	6.1	96.6

2 Synthesis of 5-FU polymer conjugate

2.1 Aminolysis in the presence of *N*-hydroxyethylacrylamide (HEA) as a Michael acceptor

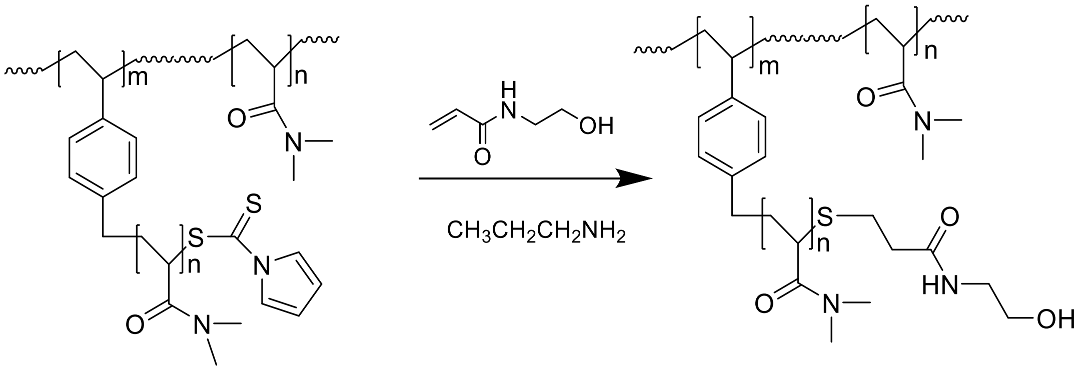

Scheme S1. One-pot aminolysis/ N-hydroxyethylacrylamide conjugation

2.2 The synthesis and attachment of vinyl-modified tetra-peptide (Gly-Leu-Phe-Gly)

2.2.1 The synthesis of vinyl-modified tetra-peptide (Gly-Leu-Phe-Gly)

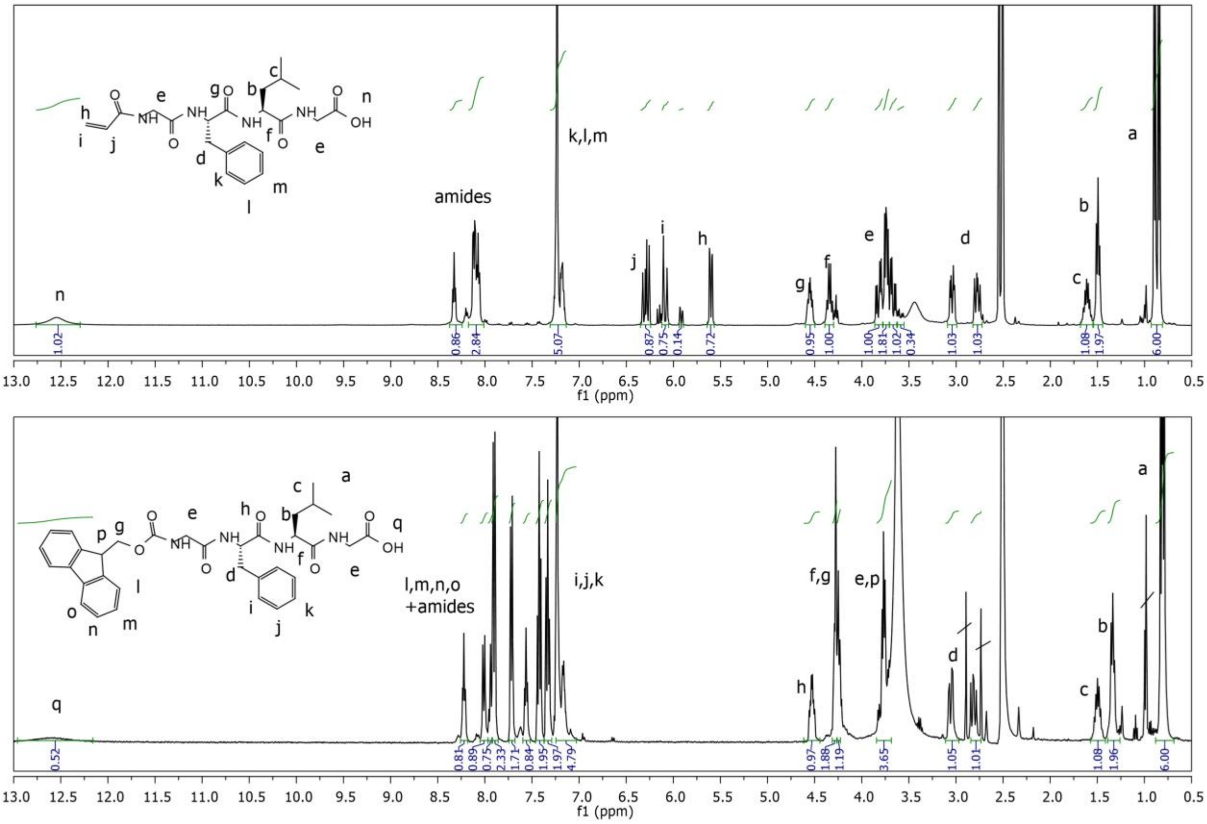

Figure S5. ^{1}H NMR of non-modified and vinyl modified peptide in $(CD_3)_2SO$

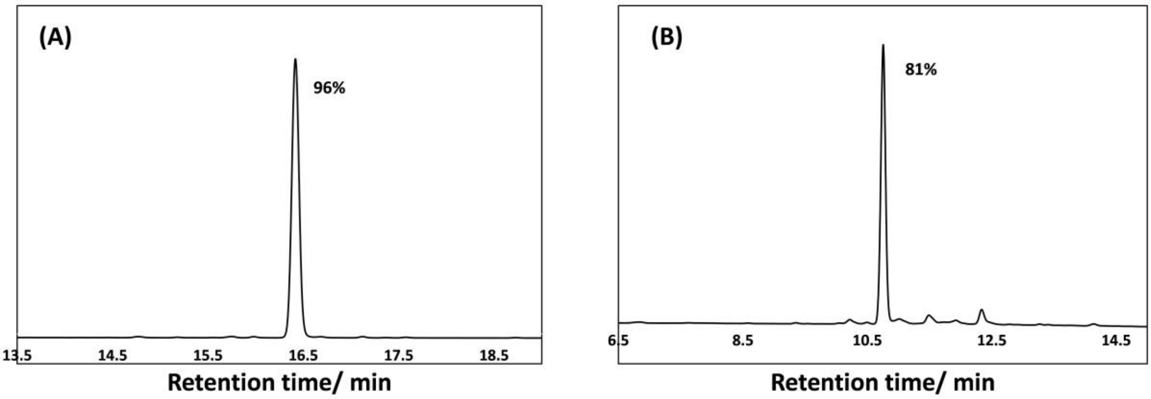

Figure S6. HPLC profiles of (A) non-modified peptide (B) vinyl-modified peptide. Column: Waters XBridge C18 250 × 4.6 mm. Mobile phase: gradient 5 to 95% acetonitrile (with 0.1% formic acid) over 20 min at a flow rate of 1 mL min^{-1}

2.2.2 The conjugation of vinyl-modified tetra-peptide (Gly-Leu-Phe-Gly) into HB-PDMA

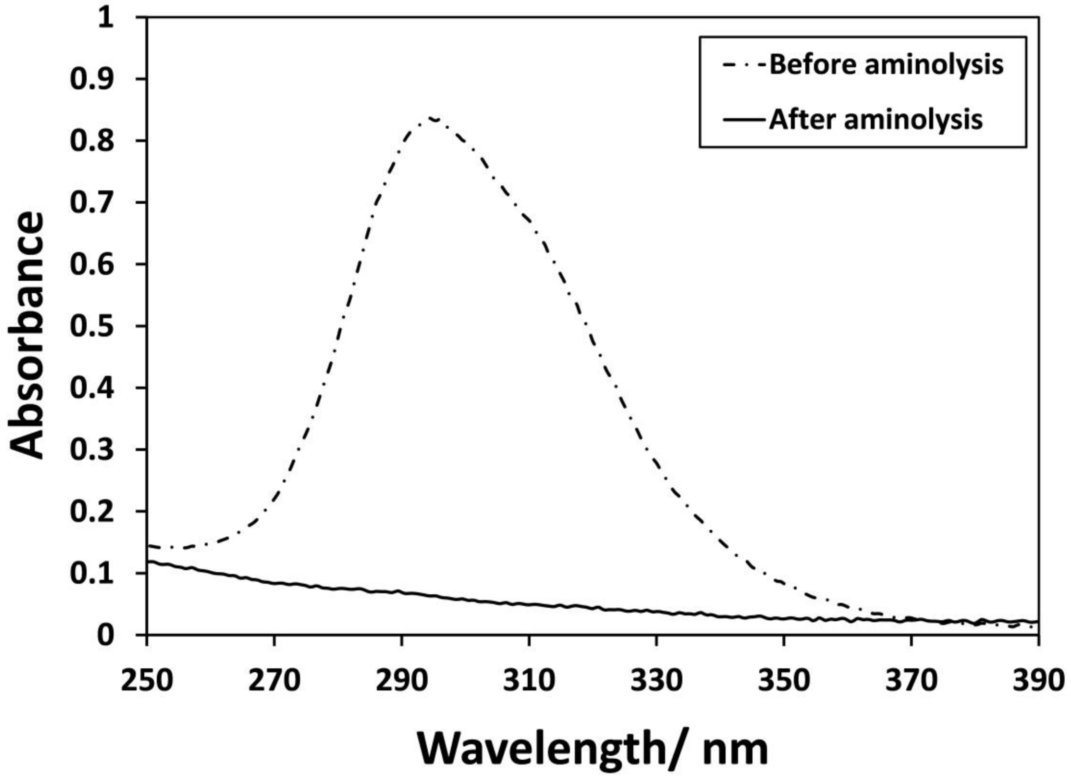

Figure S7. UV-vis absorbance spectra of HB-PDMA before and after aminolysis in water

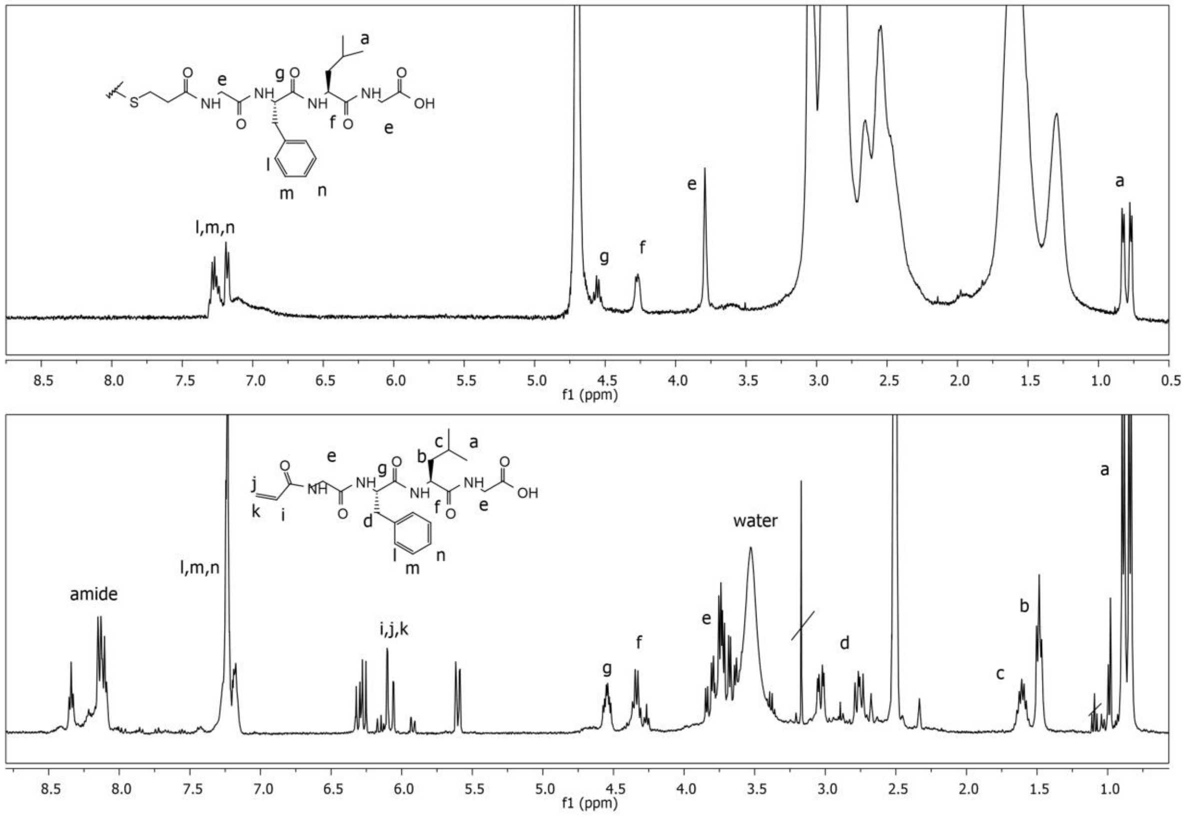

Figure S8. ^{1}H NMR spectra of HB-PDMA-Gly-Leu-Phe-Gly in D_2O and vinyl-modified tetra-peptide (Gly-Leu-Phe-Leu) in $(CD_3)_2SO$

2.3 The synthesis and attachment of the dipeptide, Leu-Gly(5-FU)

2.3.1 The synthesis of the dipeptide, Leu-Gly(5-FU)

Table S3. Cbz deprotection using various hydrogen transfer agents

Cbz-protected peptide	Hydrogen donor	Product	Comments
**Cbz-Leu-Gly(5FU)-OMe**	Cyclohexene	No reaction	
**Cbz-Leu-Gly(5FU)-OMe**	Hydrazine	The peptide was destroyed	
**Cbz-Leu-Gly(5FU)-OMe**	Formic acid	Leu-Gly(5FU)-OMe	Partial hydrogenation of pyrimidine ring
**Cbz-Leu-Gly(5FU)-OH**	1,4-cyclohexadiene	Leu-Gly(5FU)-OH	

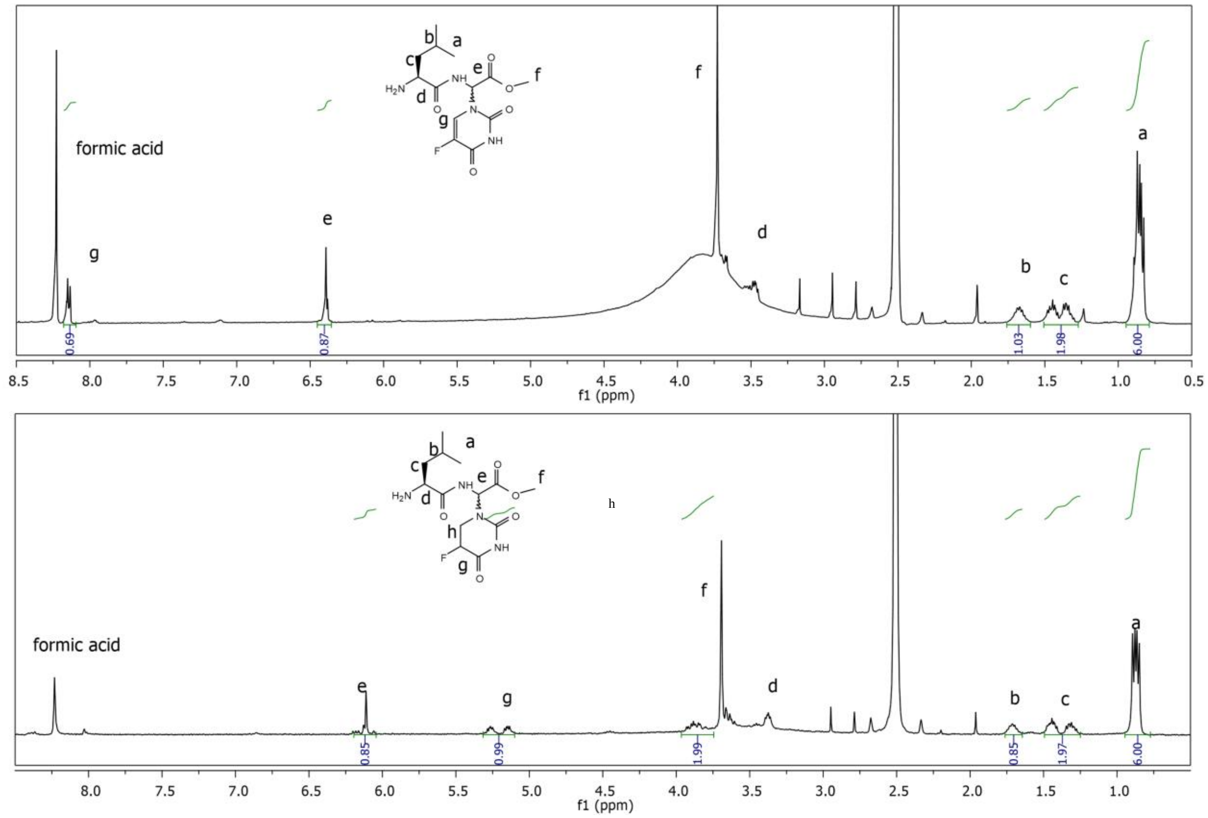

Figure S9. ^1H NMR spectrum in $(CD_3)_2SO$ of the isolated side product after the hydrogenation of 5-FU prodrug with formic acid at room temperature. The disappearance of the doublet at 8.16 ppm due to pyrimidine CH proton along with the presence of the resonance signals for CH_2-CHF protons (3.86 and 5.12– 5.26 ppm) indicates the hydrogenation of the double bond in the pyrimidine ring.

2.3.2 The conjugation of the dipeptide, Leu-Gly(5-FU)

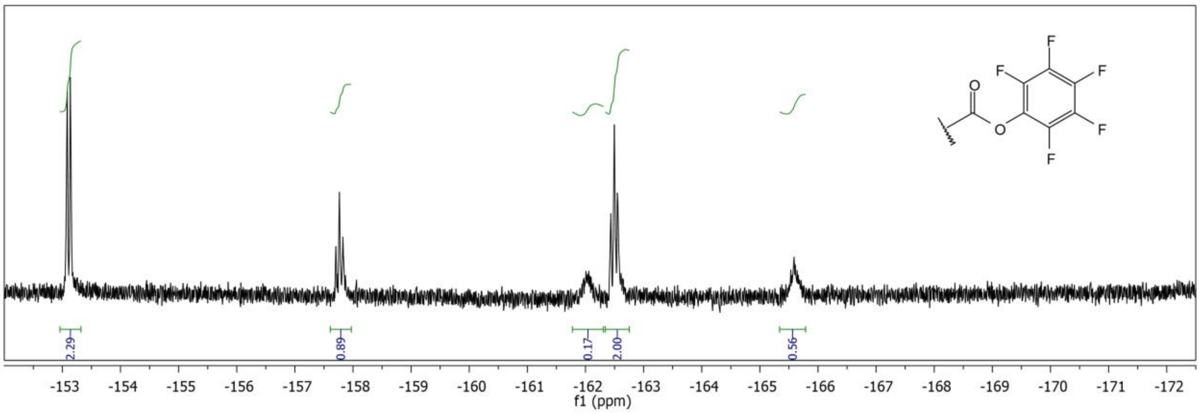

Figure S10. ^{19}F NMR of HB-PDMA-Gly-Leu-Phe-Gly with pentafluorophenyl ester end group in $(CD_3)_2SO$

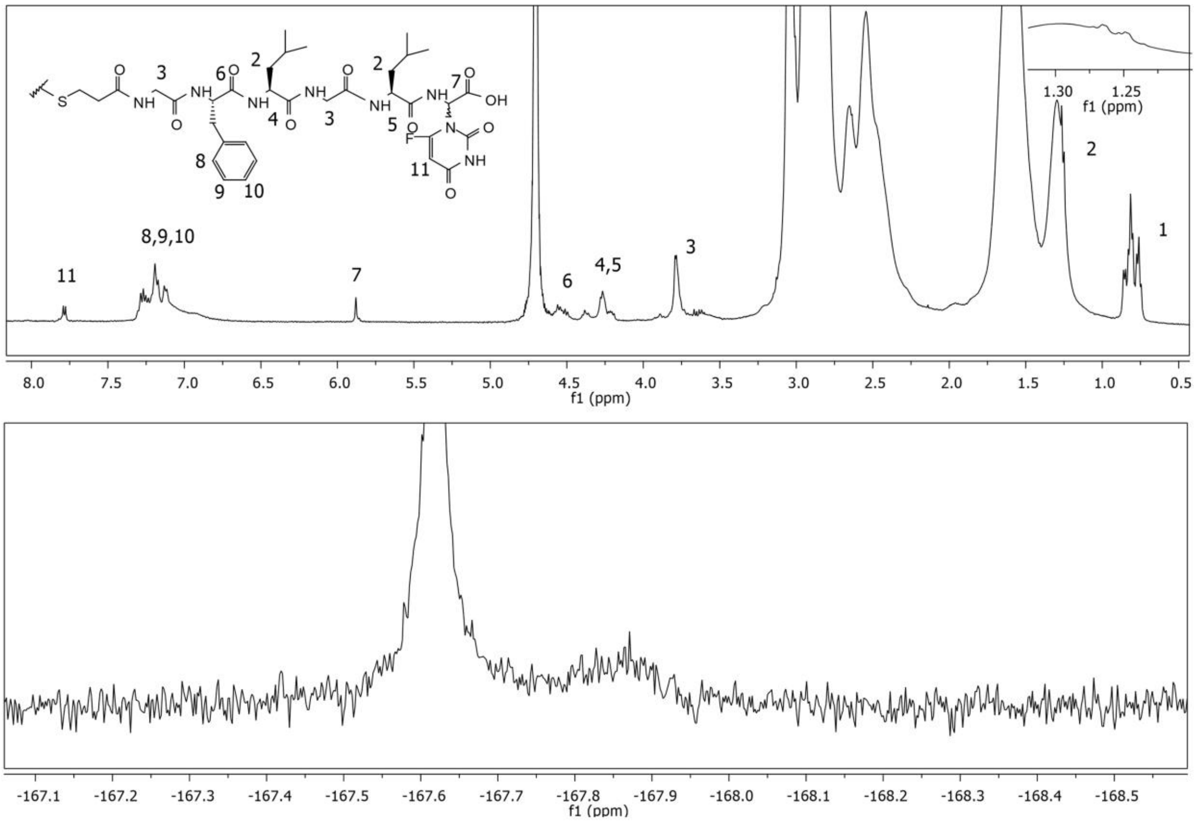

Figure S11. ^{1}H NMR and ^{19}F NMR of HB-PDMA-Gly-Leu-Phe-Gly-Leu-Gly(5-FU) in D_2O. The two signals due to the α-hydrogen of leucine at 4.21 and 4.38 ppm indicates different environments due to the formation of the dipeptide and the desired hexapeptide conjugates.

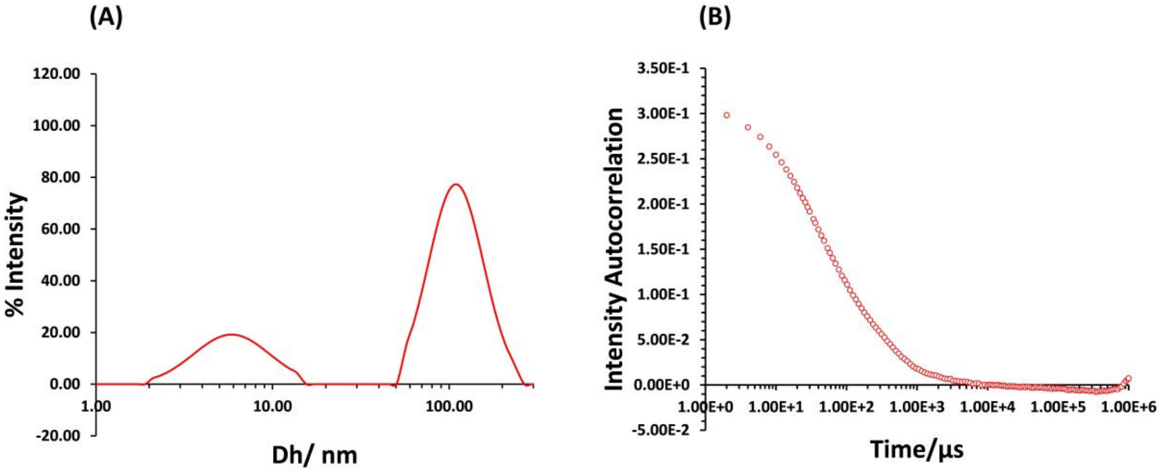

Figure S12. (A) Size distribution of the polymer peptide conjugate at pH 7.4 determined by DLS **(B)** Autocorrelation curve

3 Monitoring the release of 5-FU using ^{19}F NMR

Table S4. List of ^{19}F T_1 and T_2 relaxation times of 5-FU, 5-FU prodrug, and the polymer conjugate at pH 7.4

	T_1/ s	T_2/ s
**5-FU**	4.810	0.476
**5-FU prodrug**	1.049	0.053
**Polymer conjugate**	0.835	0.038

(A)

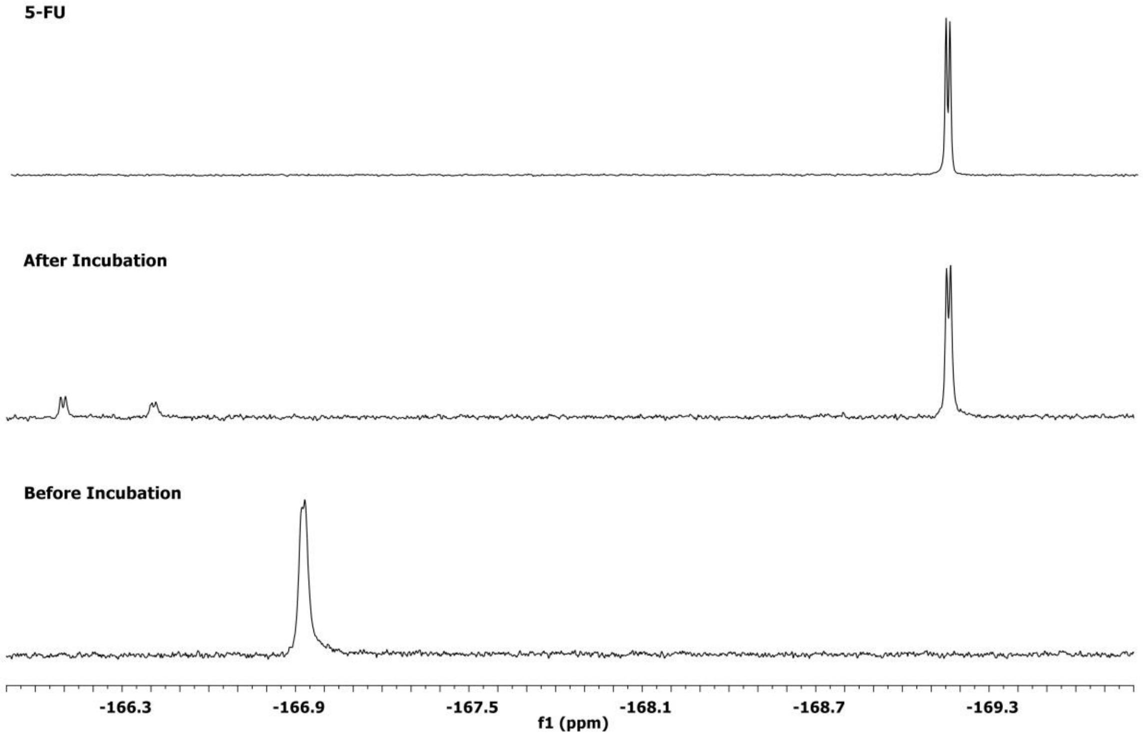

(B)

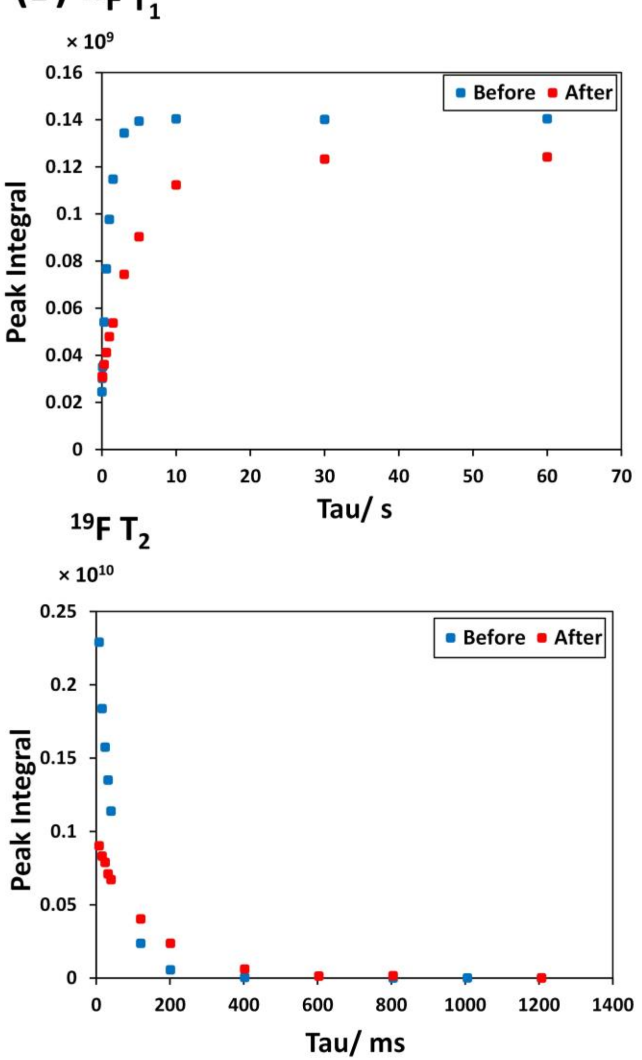

(C)

	T_1/ s	T_2/ s
**Before Incubation**	0.997	0.047
**After Incubation**	4.804	0.144

Figure S13. (A) ^{19}F NMR spectrum of L,L dipeptide before and after the treatment with S9 fraction from liver in PBS:D_2O 9:1 containing 3 mM $MgCl_2$ **(B)** ^{19}F T_1 and T_2 relaxation times curves of L,L dipeptide before and after treatment **(C)** list of ^{19}F T_1 and T_2 relaxation times of L,L dipeptide before and after the treatment
